# *SSAVE*: A tool for analysis and visualization of sleep periods using electroencephalography data

**DOI:** 10.3389/frsle.2023.1102391

**Published:** 2023-01-27

**Authors:** Amlan Talukder, Yuanyuan Li, Deryck Yeung, David M. Umbach, Zheng Fan, Leping Li

**Affiliations:** 1 Biostatistics and Computational Biology Branch, National Institute of Environmental Health Sciences, RTP, NC, United States,; 2 Department of Engineering Science, Trinity University, San Antonio, TX, United States,; 3 Division of Sleep Medicine, University of North Carolina at Chapel Hill, Chapel Hill, NC, United States,; 4 Department of Neurology, University of North Carolina at Chapel Hill, Chapel Hill, NC, United States

**Keywords:** sleep cycle, EEG, circadian rhythm, NREM, REM

## Abstract

Human sleep architecture is structured with repeated episodes of rapid-eye-movement (REM) and non-rapid-eye-movement (NREM) sleep. An overnight sleep study facilitates identification of macro and micro changes in the pattern and duration of sleep stages associated with sleep disorders and other aspects of human mental and physical health. Overnight sleep studies record, in addition to electroencephalography (EEG) and other electro-physiological signals, a sequence of sleep-stage annotations. *SSAVE*, introduced here, is open-source software that takes sleep-stage annotations and EEG signals as input, identifies and characterizes periods of NREM and REM sleep, and produces a hypnogram and its time-matched EEG spectrogram. *SSAVE* fills an important gap for the rapidly growing field of sleep medicine by providing an easy-to-use tool for sleep-period identification and visualization. *SSAVE* can be used as a Python package, a desktop standalone tool or through a web portal. All versions of the *SSAVE* tool can be found on: https://manticore.niehs.nih.gov/ssave.

## Introduction

Sleep is essential for good physical and mental health, yet physical and mental conditions also affect sleep (Krause et al., 2017; Franks and Wisden, 2021; Paller et al., 2021). In fact, sleep architecture can reflect human physiology (Ju et al., 2014; Mander et al., 2017). Sleep quantity and quality tend to differ with sex and age as well as among people who suffer from sleep disorders, stress, anxiety, neuropsychiatric disorders, or diabetes (Redline et al., 2004; Wulff et al., 2010; Ju et al., 2014; Mander et al., 2017; Högl et al., 2018).

A normal overnight sleep is structured with certain characteristics that reflect a dynamic physiological process. Sleep is broadly dichotomized into non-rapid eye movement (NREM) sleep and rapid eye movement (REM) sleep. NREM sleep is further sub-divided into three stages–N1, N2 and N3–based on the new American Academy of Sleep Medicine (AASM) rules (Moser et al., 2009). This structure is generally monitored with an in-laboratory overnight sleep study, also called a polysomnography (PSG) study. PSG records electrical signals from multiple sources; these include electroencephalography (EEG) or voltage signals from the cerebral cortex as well as signals for muscle and eye movement and heart activity. These sensor data are grouped into a sequence of temporal units; each unit, known as an “epoch,” has a fixed duration (typically 30 s). In addition, a qualified sleep technician uses data from these sources and scores each epoch as one of five sleep stages: wake (W), N1, N2, N3 or REM (R) (Berry et al., 2017). The sleep-stage annotation for each epoch is embedded together with electrical signals as part of the data arising from a PSG study.

The basic structural organization of sleep stages during an overnight sleep is referred to as sleep architecture and reflects a pattern known as the ultradian rhythm (Adamantidis et al., 2019; Goh et al., 2019). Typically, in a healthy adult, NREM and REM stages alternate throughout an overnight sleep, along with a few W stages. A sequence of epochs composed predominantly of NREM stages constitutes an NREM period or NREMP; similarly, a sequence of epochs composed predominantly of REM stages constitutes a REM period or REMP. The alternating pattern of NREMP and REMP with the duration of the respective periods varying throughout the night is typically referred to as a “sleep cycle,” although “sleep cycle” is not a precisely defined concept (Feinberg and Floyd, 1979). Normal sleep architecture in healthy young adults usually contains 4–6 sleep cycles, each consisting of an NREMP and a REMP (Hartmann, 1968; Feinberg and Floyd, 1979; McCarley, 2007; Kryger et al., 2017; Adamantidis et al., 2019; Knoop et al., 2021). The number and structure of the NREMPs and REMPs during a full night sleep exhibit patterns that can distinguish between subjects of different age groups or between healthy subjects and those with underlying sleep disorders.

Sleep stages, and consequently sleep periods, are highly associated with EEG electrical activity. EEG signals recorded as temporal sequences of voltages from multiple scalp electrodes can be re-expressed as frequencies in five primary frequency bands of interest: delta (<4 Hz), theta (4–7 Hz), alpha (8–12 Hz), beta (13–30 Hz) and gamma (>30 Hz), along with special frequency sub-bands like spindles (11–15 Hz) and saw tooth waves (2–6 Hz) (Newson and Thiagarajan 2019). A spectrogram displays the power at each frequency through time. Spectrograms contain rich information on sleep-stage transitions and ultradian features that are important for sleep disorder detection (Iber et al., 2007). The number and structure of the sleep periods and cycles during a full night’s sleep as visualized by hypnograms and spectrograms provide distinguishing patterns between subjects with different physiological conditions or mental states. Consequently, successful identification and visualization of NREMPs and REMPs can aid physicians’ diagnoses.

To our knowledge, “*SleepCycles*” (Blume and Cajochen, 2021) is the first, and currently the only, tool that uses a sequence of epoch-wise sleep-stage annotations to identify sleep cycles and their respective NREMPs and REMPs. *SleepCycles* is an R package; it outputs a hypnogram mapping the detected sleep periods to a time axis. It also provides the flexibility to choose the start of a sleep period and to set the REM period duration. Although *SleepCycles* has proved highly useful for sleep clinicians and researchers, its utility can be enhanced. As an R package, users must have some familiarity with that programming language to run it. In addition, users interested in seeing a spectrogram to complement the *SleepCycles*-generated hypnogram must familiarize themselves with spectrogram analysis and must learn and run separate software to generate the spectrogram. Finally, the rules for sleep-cycle and sleep-period detection applied by *SleepCycles*, though based on criteria adapted from seminal work by Feinberg and Floyd (1979), allow wake stages of arbitrary length within an NREMP or a REMP–a choice that conflicts with the common-sense notion that extended wakefulness should not be included as part of a sleep period.

Our goal was to develop a tool for identifying sleep periods that retained the much of the conceptual framework of *SleepCycles* but expanded its utility. We wanted a tool that was fast, versatile, easy to use, and accessible without specialized programming expertise. In addition, we wanted it to take as input either sleep-stage annotations alone or EEG data with embedded annotations; so that, with appropriate data, the tool could display both a sleep-stage hypnogram and a time-matched spectrogram. Finally, we sought to modify the rules for identifying sleep periods to address anomalies may occur during a PSG recording–such as long-duration wakefulness. Our tool, *SSAVE*, was developed in Python. We provide both web-based and desktop-based applications for *SSAVE*, including a command line version for users.

## Methods

### Overview of *SSAVE*

**FIGURE 1:**
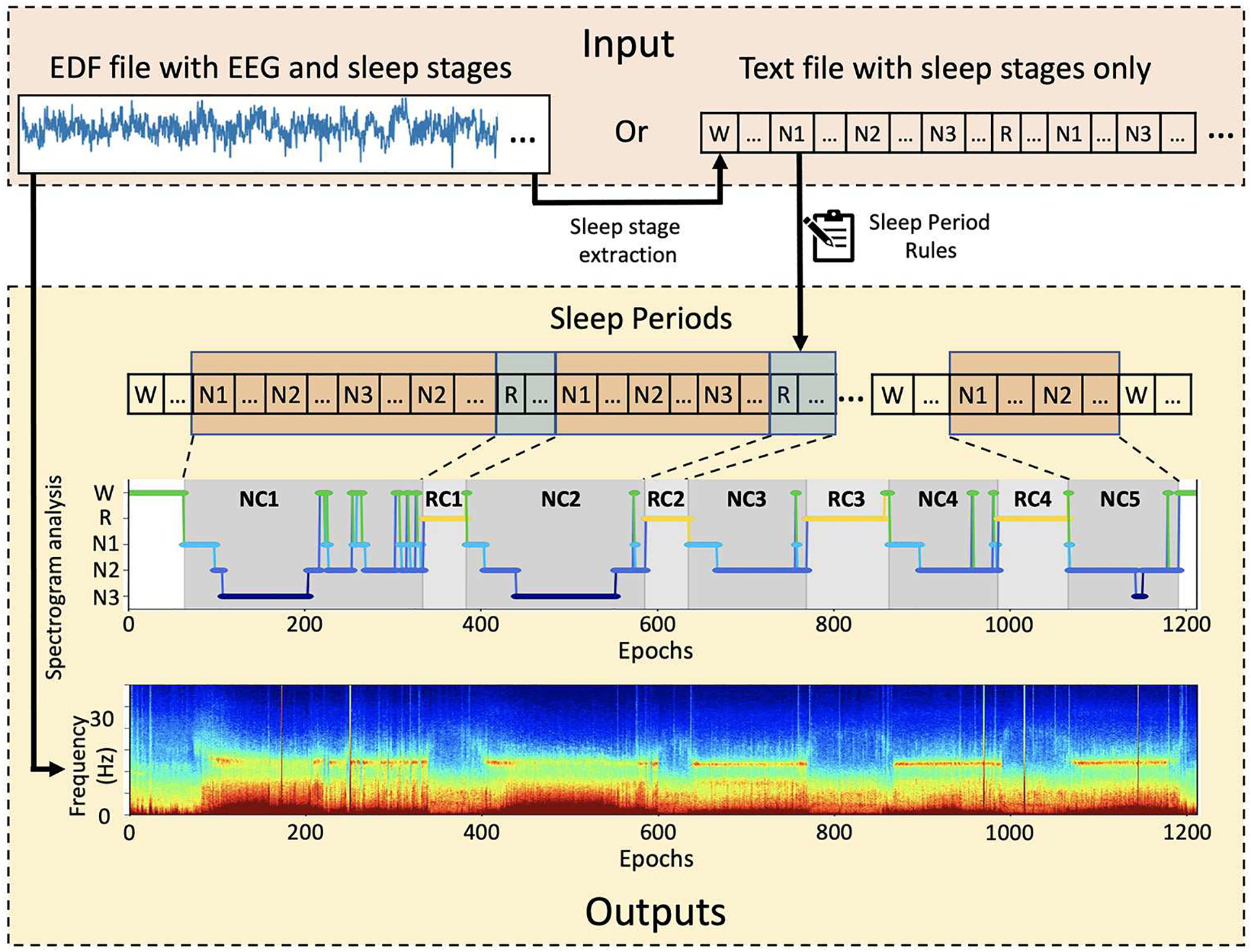
Overview of *SSAVE*. *SSAVE* takes EEG signals and sleep-stages annotations (N1, N2, N3, R, W) and outputs sleep periods as a hypnogram with different shading for NREM and REM periods (denoted as NC and RC, respectively) together with a time-matched spectrogram.

*SSAVE* was designed to identify sleep periods from the sleep-stage annotations that are embedded with EEG signals in data files in the EDF (European Data Format) format. The EDF file formats can be vendor dependent. SSAVE extracts epoch-based sleep stages from the embedded annotations provided by board-certified sleep technicians/doctors using all polysomnography data and employs our revised rules (Supplementary Table S1) (Feinberg and Floyd, 1979; Blume and Cajochen, 2021) to identify sleep periods. *SSAVE* outputs the hypnogram as a JPEG image and a listing of epoch-based sleep stages and sleep periods in text format. If EEG data are the input, *SSAVE* uses the EEG signals to automatically generate a multitaper spectrogram (Prerau et al., 2017) that is time-matched to the hypnogram. If sleep-stage annotations are not available in the EDF file, *SSAVE* can generate only the spectrogram but neither the sleep periods nor the corresponding the hypnogram. Alternatively, *SSAVE* can input user provided sleep annotations in text format and outputs only the hypnogram and listing (Figure 1).

### Processing of input data

With EEG data as input, *SSAVE* simultaneously divides the EEG signal data and sleep-stage annotations embedded in the EDF data into epochs commensurate with the annotation timestamps. An epoch size of 30 s is the default, but that value can be changed by the user. Any epochs without sleep-stage annotation, are removed from both the annotations and the signal data. *SSAVE* then constructs a hypnogram (a graph representing the stages of sleep as a function of time) using the annotations and identifies NREMPs and REMPs using its identification rules. It also generates a spectrogram using the EEG signal data aligned with the timestamps of the hypnogram. When a text file provides sleep-stage annotations as the only input, similar to *SleepCycles*, *SSAVE* constructs the hypnograms and identifies the sleep periods without generating the spectrogram. *SSAVE* currently requires sleep-stage annotations to identify sleep periods and construct a hypnogram.

### Overview of sleep-period characteristics

An NREMP begins with an N1 or N2 stage, whichever appears first. A REMP begins with an R stage. The NREMP must be at least 15 min long. No minimum duration is required for the first REMP. All subsequent REMPs must be longer than 5 min. Short duration (i.e., <5 min) R and W stages are allowed within NREMPs. Similarly, short duration of NREM and W stages are allowed within REMPs. NREMPs longer than 120 min are split with user feedback. Detailed rules are provided below.

### Rules for identifying sleep periods

*SSAVE* scans from low to high timestamps of the annotation epochs to detect a sleep period using a set of rules (Supplementary Table S1). We adopted most of the traditional rules used previously (Feinberg and Floyd, 1979; Blume and Cajochen, 2021) with the following five modifications.

1. *SSAVE* does not explicitly define sleep cycles; instead, it defines the two types of component sleep periods, NREMP and REMP, separately. This approach differs somewhat from that of *SleepCycle* which considers a pair of NREMP and REMP together as a sleep cycle (Blume and Cajochen, 2021). This approach of focusing on individual sleep periods is useful. For example, the first NREMP is often not accompanied by a REMP for young adults. Moreover, NREMP and REMP have distinct characteristics such as duration and frequency, especially for younger subjects or those with sleep disorders (Ohayon et al., 2004; Galland et al., 2012). Treating NREMP and REMP separately, instead of combining consecutive pairs into cycles, enables one to obtain distinct features for each NREMP and REMP. This emphasis on sleep periods is also in accord with work suggesting that NREM and REM sleep have distinct regulation mechanisms (Le Bon et al., 2002).
2. Like *SleepCycles*, *SSAVE* sets the minimum and maximum durations of an NREMP (excluding any internal W stages) to be 15 and 120 min, respectively. For an NREMP longer than 120 min, both tools accommodate splitting the NREMP (details below). This feature is important especially when a REMP does not occur between two NREMPs (Blume and Cajochen, 2021). Neither tool sets a maximum duration for REMPs. The minimum duration for a REMP is 5 min, although this restriction does not apply to the first REMP. If an NREMP is <15 min long or a REMP (except the first one) is <5 min long, *SSAVE* merges it into the adjacent respective sleep periods.
3. The first NREMP ends upon encountering an R stage. All remaining NREMPs and all REMPs end, respectively, upon encountering consecutive REM and NREM stages of at least 5 min duration. There may be occasions when a person wakes up from sleep in the middle of an NREMP (REMP) for at least 5 min and falls back to sleep. In this scenario, *SleepCycles* considers the whole period as one NREMP (REMP). In contrast, *SSAVE* breaks the NREMP (REMP) into two NREMPs (REMPs) while omitting the W stages.
4. In *SSAVE*, an NREMP may contain W stages if and only if the W stages are <5 consecutive minutes long. When it encounters consecutive W stages lasting at least 5 min, *SSAVE* ends the current sleep period, removes the W stages, and begins a new sleep period. This rule allows *SSAVE* to properly handle EEG data with frequent W stages [e.g., EEG data for insomnia patients where the subject may wake up frequently in the middle of sleep cycles (Wei et al., 2017)]. In *SleepCycles*, all NREMPs may contain W stages regardless of their duration.
5. An NREMP that is longer than 120 min is split at the first point of either arousal or “lightening of sleep”; but *SSAVE* and *SleepCycles* choose splitting points differently. *SleepCycles* splits at the end of the first N3 stage after at least 12 consecutive minutes of N1, N2 or W stages. *SSAVE*, instead, splits the NREMP at an “arousal” point defined by consecutive W stages exceeding 1 min or at a “lightening of sleep” point defined by consecutive N1 stages exceeding 3 min. Because these definitions may admit multiple splitting positions for a long NREMP, the user is provided the flexibility to choose the splitting positions by selecting the appropriate epoch number (in the web and desktop versions) or by typing it (in the command line interface). Note that a REMP does not have any maximum duration, thus, is never split.

Lastly, sleep-stage annotation may begin before sleep commences and continue after sleep ends; the annotation file typically includes these superfluous W stages. Both tools ignore W stages before the first NREMP and W stages after the last REMP or NREMP. Because newborns have unique sleep structures, these rules are more appropriate for individuals at least 1 year old.

### Multitaper spectrogram

*SSAVE* provides a multitaper spectrogram aligned with the sleep hypnogram in the visualization plot. A spectrogram is a frequency domain representation of EEG signal power through time as a heatmap. Its X-axis shows the time (in epochs), its Y-axis shows the frequency, and color indicates the relative signal power. *SSAVE* aligns its multitaper spectrogram with the epochs of the sleep hypnogram (Supplementary Figure S1, Section 1). This alignment can help users to identify useful sleep band features (e.g., alpha, beta, gamma, delta, theta waves), and sleep spindles (Paller et al., 2021) and map them to sleep periods.

### Implementation

*SSAVE* was developed with Python version 3. It can be imported either as a Python package or run as a standalone desktop application. We have also developed a web-based portal so that users can directly access the tool using a web browser. The web-based portal can be slower than the package or the desktop version, as it requires the user to upload an input file to the *SSAVE* server (https://manticore.niehs.nih.gov/ssave).

## Results

### User interface

The web-based and the desktop-based applications present a similar user interface (Supplementary Figures S2–S6). Initially, the user will see a home window prompting for either one of the two data inputs: an EDF file containing EEG signals with embedded sleep-stage annotations; or a text file of only sleep-stage annotations having two columns, epoch number and sleep stage (“W,” “N1,” “N2,” “N3,” “R”) (Supplementary Figure S2A). The desktop-based and the command-line versions take the output directory as an additional input. Data are loaded when the “Load” button is clicked (Supplementary Figure S2B). The “Execute” button allows *SSAVE* to extract the sleep periods and generate the visualization figure and a listing of epoch-based sleep stages and identified sleep periods (Supplementary Figure S2C). The outputs are shown in the user interface under the “Visualization” and “Sleep Periods and Stages” tabs (Supplementary Figure S2D) and can be downloaded (See “Output” below).

*SSAVE* allows a user to split long NREMP (Supplementary Figure S3). When a NREMP is longer than 120 min, *SSAVE* searches for suitable epoch positions to split the period based on the arousal or lightening-of-sleep criteria mentioned earlier. If *SSAVE* finds such epoch positions, an additional tab “NREMP cut points” will appear (Supplementary Figure S3A). This tab shows a table of suitable epochs at which the long NREMP may be cut (Supplementary Figure S3B). User can select a cut option and click “Execute” to apply it (Supplementary Figure S3C). Once execution completes, the new outputs will show up with the split applied (Supplementary Figure S3D).

*SSAVE* uses default settings (Supplementary Table S2) to analyze the sleep periods, but the user can change the defaults in the settings window (Supplementary Figure S4). The settings window opens by clicking the “Configure” button at the home window after the input data are loaded. This window has four tabs corresponding to four categories of settings. In the “Select Channels” panel, the user can select their preferred EEG channels for analysis. The default settings contain eight channels: F3, F4, C3, C4, O1, O2, M1 and M2 (Supplementary Figure S4A). The “Select Sleep Stage” panel helps user to assign the proper annotations for the five sleep stages (Supplementary Figure S4B). User can also apply filters to remove noise and irrelevant epochs from the data using the “Set Filters” panel (Supplementary Figure S4C). The filters include two types of frequency filters (notch and bandpass); and three epoch filters (to remove epochs with maximum amplitude, flat signals, and unwanted annotations that do not bear useful information, e.g., bathroom breaks). The “Set Epoch Size” panel allows users to change the epoch size if the data provided as input was constructed with an epoch size different from 30 s (Supplementary Figure S4D). The user needs to re-run the analyses after changing the settings by clicking the “Execute” button at the home window. The filters can be optionally applied by checking the “Apply Filters” checkbox and then clicking the “Execute” button. All other settings will be automatically applied once the “Execute” button is clicked.

For machines without desktop support, *SSAVE* can be used as a command-line application. After running the tool, the user is prompted for the same inputs as above.

The *SSAVE* file “app_desktop.py” represents the desktop or command-line front end of the tool. The “utils.py” file contains the default settings of the tool. The “visualize_sleep.py” file can be imported as a Python package to use for further analysis.

### Output

*SSAVE* generates four outputs: the visualization figure (both hypnogram and spectrogram, or hypnogram alone, depending on input), a listing of epoch-based sleep stages, a listing of the sleep periods identified, and spectrogram data matrix containing epoch, frequency band and channel-wise EEG signal power data written to a NumPy file with “.npz” extension. The dimension of the spectrogram data matrix is (number of epochs × 901 × number of channels) (see Supplementary Method). The desktop-based and the command-line versions save output files in the user-specified output directory. The web-based application requires the user to download any files to be saved.

### Key differences between *SSAVE* and *SleepCycles*

*SSAVE* mimics *SleepCycles* (Blume and Cajochen, 2021) in concept but offers several notable advantages. First, *SSAVE* was developed to be used easily in multiple platforms for users with or without programming skills. The web version provides a convenient platform for all users. *SleepCycles* requires familiarity with the R programming language. Second, both *SleepCycles* and *SSAVE* takes sleep stage annotations in text format as input, but only *SSAVE* takes EEG data with embedded sleep stage annotations as input. When EEG data are input, *SSAVE* outputs not only a hypnogram with the identified sleep periods but also the time-matched spectrogram. The spectrogram provides power in spectral bands that are characteristic of sleep periods. *SleepCycles* provides only a hypnogram. Third, *SSAVE* uses revised rules for identifying sleep periods to avoid including long consecutive W stages within any NREMP or REMP, in contrast to *SleepCycles*. Below, we provide three examples to illustrate these key differences.

**FIGURE 2:**
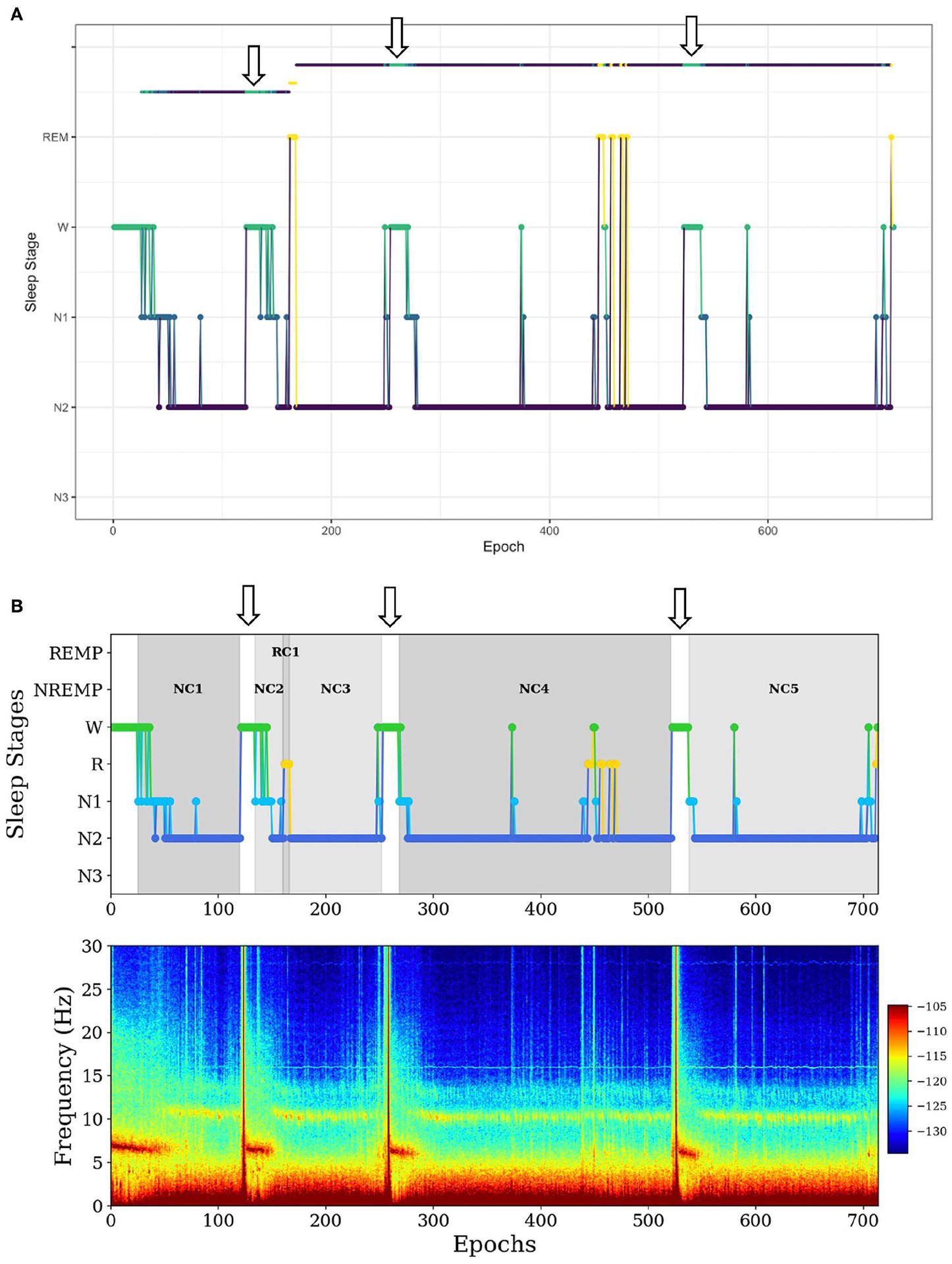
Example of difference between *SleepCycles* **(A)** and *SSAVE* **(B)** in handling consecutive wake stages of long duration within a sleep period. The wake stages are indicated by arrows. *SSAVE* removes awakenings from the sleep period, while *SleepCycles* includes them. In this example, *SleepCycles* identifies two NREM periods and one REM period whereas *SSAVE* identifies five NREM periods and one REM period by splitting the first and second NREM periods into two and three NREM periods, respectively.

In the first example, *SleepCycles* fails to exclude a long period (more than 5 min) of wakefulness in a sleep period (Figure 2A). In contrast, *SSAVE* breaks the current NREMP or REMP and starts a new one after excluding consecutive W stages (Figure 2B). As a result, *SSAVE* allows consecutive W stages within a sleep period only if their duration is <5 min (10 epochs). This choice allows an NREMP or REMP to contain brief intervals of arousal or lightening of sleep without containing intervals where a subject is likely fully awake.

**FIGURE 3:**
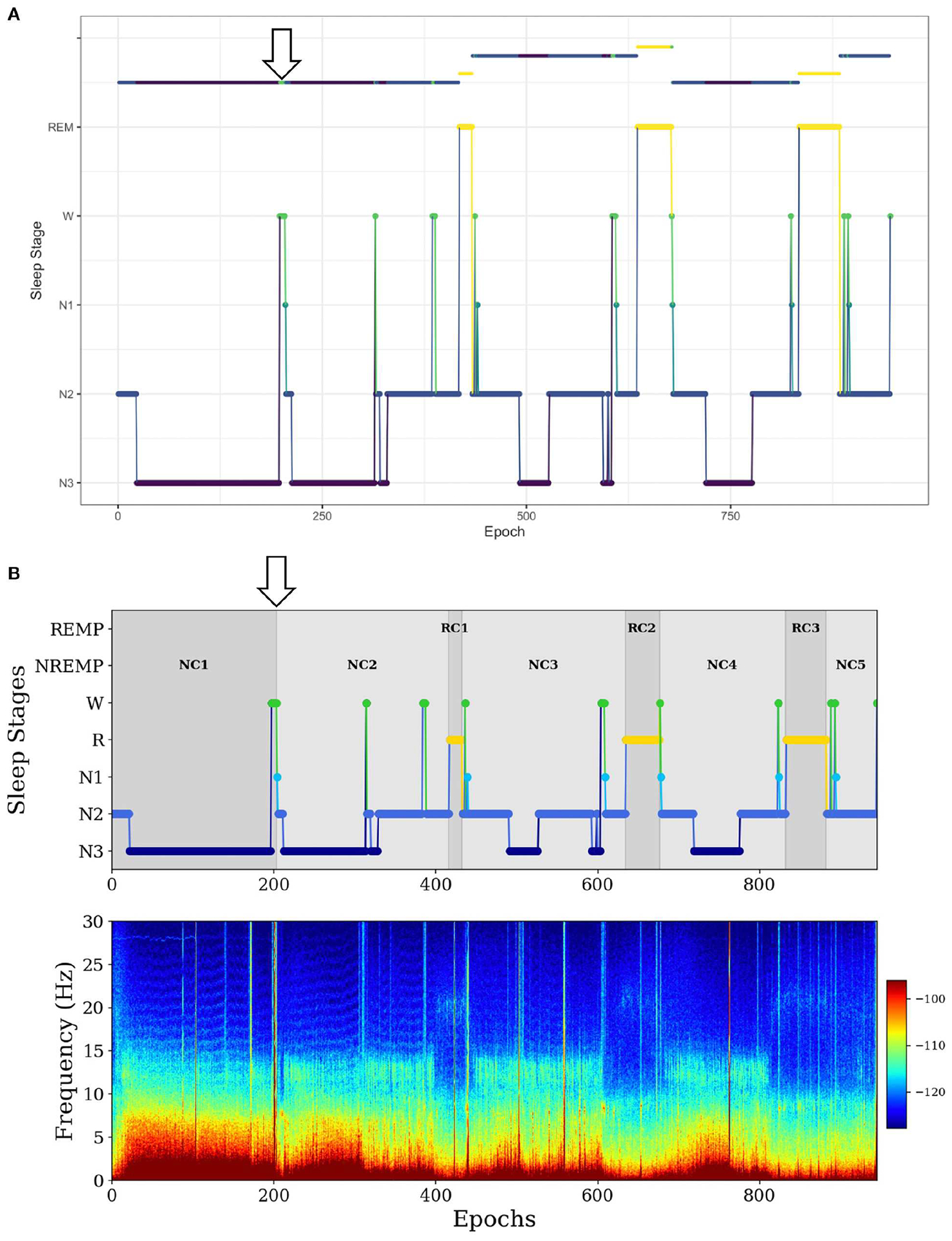
Example of difference between *SleepCycles* **(A)** and *SSAVE* **(B)** in handling the first long NREM period. In this example, *SSAVE* finds a reasonable position to split the long NREM period (indicated by arrow), whereas *SleepCycles’* stringent splitting criterion does not.

In the second example, for a long NREMP (more than 2 h), *SleepCycles* searches for the first N3 stage preceded by at least 12 min of consecutive N1, N2 or W stages and then splits the NREMP at the beginning of the N3 stage, resulting in two consecutive NREMPs (Figure 3A). Instead, *SSAVE* searches for the first N3 stage immediately preceded either by more than 1 min of consecutive W stages and splits the NREMP at the beginning of the N3 stage. Alternatively, it searches the for the first N3 stage following more than 3 min of consecutive of N1 stages and splits the NREMP at the beginning of the first N1 stage (Figure 3B). Because W or N1 stages precede deep sleep, *SSAVE*’s rule provides reasonable place to split a long NREMP.

In the third example, *SSAVE* breaks a long sleep period, either NREMP or REMP, upon encountering more than 5 min of consecutive W stages and starts a new sleep period after their removal. This process divides long sleep periods, so that two consecutive NREMPs or two consecutive REMPs are possible. This possibility is one reason why *SSAVE* does not explicitly define a sleep cycle as an NREMP followed by a REMP.

## Discussion

Sleep is structured. Infants have different sleep structure compared to children and adults. For children and adults, early sleep normally starts with the N1 stage, followed by the N2 stage, then the N3 stage (all NREM sleep). N1 is usually short lasting 5–10 min. In N1, both the body and brain slow down to prepare for a deeper sleep. In N2, further relaxation occurs with heart rate and breathing slowing down and muscle relaxed and body temperature dropping. N3 is the deep sleep in which both muscles and eyes are fully relaxed, and brain waves are largely slow waves (delta). Later in sleep, REM sleep begins to appear after NREM sleep. Traditionally, the consecutive occurrence of an NREMP and a REMP defines one sleep cycle (Feinberg and Floyd, 1979). Traditional sleep cycles–i.e., alternating NREMP and REMP–are more common in healthy children and young adults.

For those with sleep disorders or other medical conditions, normal sleep structure is usually disrupted. For example, the duration of an NREMP can be very long, making it hard to judge whether it should be one long or two shorter periods. Frequently, a REMP is too short (<5 min) to be considered part of an alternating cycle. Sometimes, an NREMP consists of only one or two of the three NREM stages. Both NREM and REM sleep can often be interrupted by many mini-arousals (wake stages lasting at least 1 min). These examples represent just a few of the challenges for identifying sleep periods or sleep cycles; in addition, identification must cope with the heterogeneity and noise present in the recorded polysomnography (PSG) data.

Overnight PSG recordings consist of electrophysiology data over many hours of sleep; these data provide information on overall sleep architecture including sleep periods. Clinicians typically use the clinically obtained shorter length PSG data and the associated hypnograms for the diagnosis of sleep-related diseases such as sleep apnea. The vast amount of the PSG data generated by an overnight sleep study are often underutilized by clinicians. For example, hypnograms only provide an overview of sleep stages and their transitions throughout the night; however, hypnograms do not include other potentially useful information such as sleep period transitions and ultradian features such as delta power and spindle density.

Here, we modestly revised previously published rules for identifying NREMP and REMP (Feinberg and Floyd, 1979; Merica and Gaillard, 1986; Blume and Cajochen, 2021) and developed user-friend software for multiple platforms. Our tool can generate and display on the same time axis both the hypnogram with sleep stages and periods and the multitaper spectrogram (Prerau et al., 2017) extracted from the raw EEG signal. The hypnogram and spectrogram are temporally aligned to provide a better comparative visualization for the user. Along with the facilities provided by *SleepCycles*, *SSAVE* provides additional flexibilities to the user, e.g., choosing of EEG channels for the spectrogram, choosing appropriate sleep annotations for the five sleep stages, applying frequency and epoch filters to the input data.

*SSAVE* applies general rules to the epoch-specific sleep stage annotations to identify sleep periods. These rules may fail to unambiguously delineate sleep periods for some subjects, however. Nevertheless, *SSAVE* will attempt to maintain fidelity to the rules in determining NREMP and REMP. In certain cases, where the identification of sleep periods is ambiguous, such as when multiple split options exist for a long NREMP, *SSAVE* will let the user choose.

Because sleep is structured, changes in or disruptions in that structure reflect underlying physiological changes. Thus, identification of sleep periods provides valuable information about health and development. For example, for healthy individuals, a REMP is typically absent or very short early in sleep, but REMP duration increases gradually as the sleep progresses (Aserinsky and Kleitman, 1953, 1955). In contrast, patients with REM behavior disorders often have a REMP early in sleep (Högl et al., 2018). The duration of NREMPs is longer in earlier cycles, however, and gradually decreases later in sleep (Feinberg and Floyd, 1979). The sleep periods identified by *SSAVE* with their accompanying visualization can help clinicians distinguish a subject with a disease from a healthy one visually.

*SSAVE* provides a unified interface for the identification and visualization of sleep stages and sleep periods throughout a subject’s entire recorded sleep period. The multitaper spectrogram associated with the hypnogram will help identify frequency-related features associated with different sleep stages including power in separate frequency bands (e.g., alpha, beta, gamma, delta, theta etc.), sleep spindles, and k-complexes. The multitaper method that we have implemented is the most widely accepted approach for generating spectrograms to date; it generates a spectrogram where the desired frequency components are isolated with high resolution (Prerau et al., 2017).

In summary, we developed a user-friendly software tool, *SSAVE*, for identification and characterization of NREMP and REMP using either PSG EEG data with embedded sleep-stage annotations or with the annotation data alone. For PSG EEG data, *SSAVE* generates a spectrogram for visualization in addition to a hypnogram. We also provide a web version of the tool along with the desktop version to allow occasional use of the tool without requiring installation. We also kept the functionality of the tool separate from its user interface, thereby allowing users integrate the functionality as a Python package. We also improved the rules for sleep-period identification. We envision that *SSAVE* will facilitate the exploration and utilization of the rich electrophysiology data available from overnight PSG studies for better understanding of sleep architecture and brain function.

## Supplementary Material

Supplemental materials

## Data Availability

The original contributions presented in the study are included in the article/Supplementary material, further inquiries can be directed to the corresponding authors.
