# Supplementary material for: SSAVE: A tool for analysis and visualization of sleep periods using electroencephalography data
Source: Front Sleep. 2023 Jan 27;2:1102391. doi: 10.3389/frsle.2023.1102391 (PMC10358288; doi:10.3389/frsle.2023.1102391)
Supplement: Supplementary file 1 [file Data_Sheet_1.docx]

**Supplementary material for**

***SSAVE*: A tool for Analysis and Visualization of Sleep Periods using Electroencephalography Data**

# Supplementary Methods

## Multi-taper Spectrogram

The following steps are required to create a multi-taper spectrogram (Prerau, Brown et al. 2017),

(1) generate a set of DPSS tapers,

(2) estimate a single-taper spectrum for each piece of tapered data and

(3) compute the mean of the single-taper spectra.

Given a specific size of the signal window and frequency resolution, the number of DPSS tapers required to create the spectrogram, can be calculated using the following equation (Prerau, Brown et al. 2017).

$N$ = size of the data window (in sec)

$\Delta f$ = frequency resolution = minimum distance between frequency peaks

$TW$ = half bandwidth = $(N* \Delta f)/2$

$L$ = number of DPSS tapers = $\left\lfloor2* TW \right\rfloor- 1$

To generate the spectrogram, we used the *time_frequency.psd_array_multitaper* function of MNE python package with $N=$ epoch size, ∆f = 2/(epoch size/2). These values were used to set the half bandwidth $TW = 2$. So, the number of DPSS tapers used to create the spectrogram was, $L = 3$.

The spectrogram contains the power at different frequency bands for each epoch and channel. For each epoch and channel, the power is calculated from 0 to 30Hz with 1Hz interval; each interval having 30 frequency bands; plus 1 more band after 30Hz. So, there are total (30x30 +1) = 901 frequency bands, taken with $TW=$ 2 sec data windows. The overall dimension of the spectrogram matrix is (number of epochs x 901 x number of channels).

# Supplementary Tables and Figures

| **Table S1:** Comparisons of rules for identifying sleep periods between *SleepCycles* and *SSAVE*. Rules or parts of rules common to both tools span both columns. Changes in *SSAVE* are in **bold**.   \| **Category** \| ***SleepCycles*** \| ***SSAVE*** \| \| --- \| --- \| --- \| \| Definition of a sleep period \| An NREMP followed by a REMP defines one sleep cycle. If the first REMP is absent, the preceding NREMP alone defines the first cycle. \| **Either an NREMP alone or a REMP alone defines one sleep period. (Cycles are not explicitly defined.)** \| \| Beginning of a sleep period \| An NREMP starts with N1 by default. If N1 stage is absent, N2 is the start of an NREMP. \| \| \| REMP starts with an R stage. \| \| \| Duration of a sleep period \| NREMP has a minimum duration of 15 min.  If an NREMP is longer than 120 minutes (excluding W stages), the user is offered split options. \| \| \| For the first REMP, no minimum duration. For other REMPs, the minimum duration is 5 min. No maximum duration for REMP. \| \| \| End of a sleep period \| The first NREMP ends when at least one R stage is encountered.  The other NREMPs end when at least 5 min of consecutive R stages are encountered. \| \| \|  \| **or when at least 5 min of consecutive W stages are encountered** \| \| REMP ends when at least 5 minutes of any N1, N2, or N3 stages are encountered. \| \| \|  \| **or when at least 5 min of consecutive W stages are encountered.** \| \| Sleep stages in a sleep period \| All the NREMP may contain N1, N2, N3, or W stages. \| \| \|  \| **if the consecutive W stages are less than 5 min long.** \| \| The first NREMP does not contain an R stage.  The other NREMPs may contain consecutive R stages for less than 5 min. \| \| \| REMP contains R and W stages and N1, N2, and N3 stages \| \| \| lasting for less than 5 min \| **only if the W stages are less than 5 consecutive min long and any consecutive N1, N2, and N3 stages are less than 5 min long.** \| \| Splitting a long sleep period \| An NREMP longer than 120 minutes (excluding W stages) is split \| \| \| at the first N3 stage which follows at least 12 min of any other sleep stages (N1 or N2 or W) \| **when the NREMP contains more than 1 min of consecutive W stages or more than 3 min of consecutive N1 stages.** \| \| REMP stage does not have any maximum duration and thus is not split. \| \| \| Wake stages \|  \| **Excludes consecutive W stages of 5 min or longer.** \| \| Excludes W stages at the beginning and end of a sleep period. \| \| |
| --- | --- | --- | --- | --- | --- | --- | --- | --- | --- | --- | --- | --- | --- | --- | --- | --- | --- | --- | --- | --- | --- | --- | --- | --- | --- | --- | --- | --- | --- | --- | --- | --- | --- | --- | --- | --- | --- | --- | --- | --- | --- | --- | --- | --- | --- | --- | --- | --- |

| **Table S2:** Default settings used by *SSAVE*.   \| **Category** \| **Settings** \| \| --- \| --- \| \| Sleep Stages \| W = “Sleep stage W” \| \| REM = “Sleep stage R” \| \| N1 = “Sleep stage N1” or “Sleep stage 1” \| \| N2 = “Sleep stage N2” or “Sleep stage 2” \| \| N3 = “Sleep stage N3” or “Sleep stage 3” or “Sleep stage 4” \| \| Channels \| F3, F4, C3, C4, O1, O2, M1, M2 \| \| Filters \| Notch frequency = 60 Hz \| \| Bandpass frequency range = 0.05 Hz to 30 Hz \| \| Maximum amplitude = 500 microvolt \| \| Flat signal duration = 5 sec \| \| Epoch size \| 30 seconds \| |
| --- | --- | --- | --- | --- | --- | --- | --- | --- | --- | --- | --- | --- | --- | --- | --- | --- | --- |

**
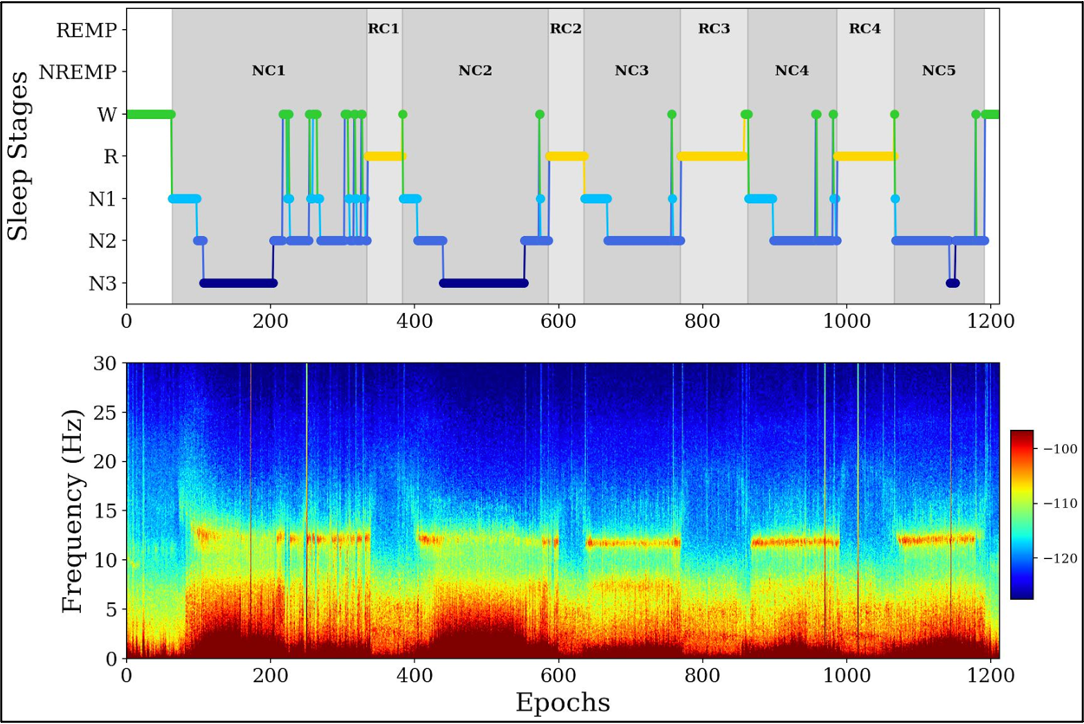
**

**Figure S1:** Example of a paired hypnogram and spectrogram generated by *SSAVE*. The hypnogram at the top shows the annotations of the five sleep stages for each epoch; sleep stages are distinguished by color and being plotted at distinct vertical positions while the NREMP and REMP sleep periods that *SSAVE* identified are distinguished by differentially shaded vertical bands. The spectrogram at the bottom is extracted from the EEG signal data for each epoch.

| **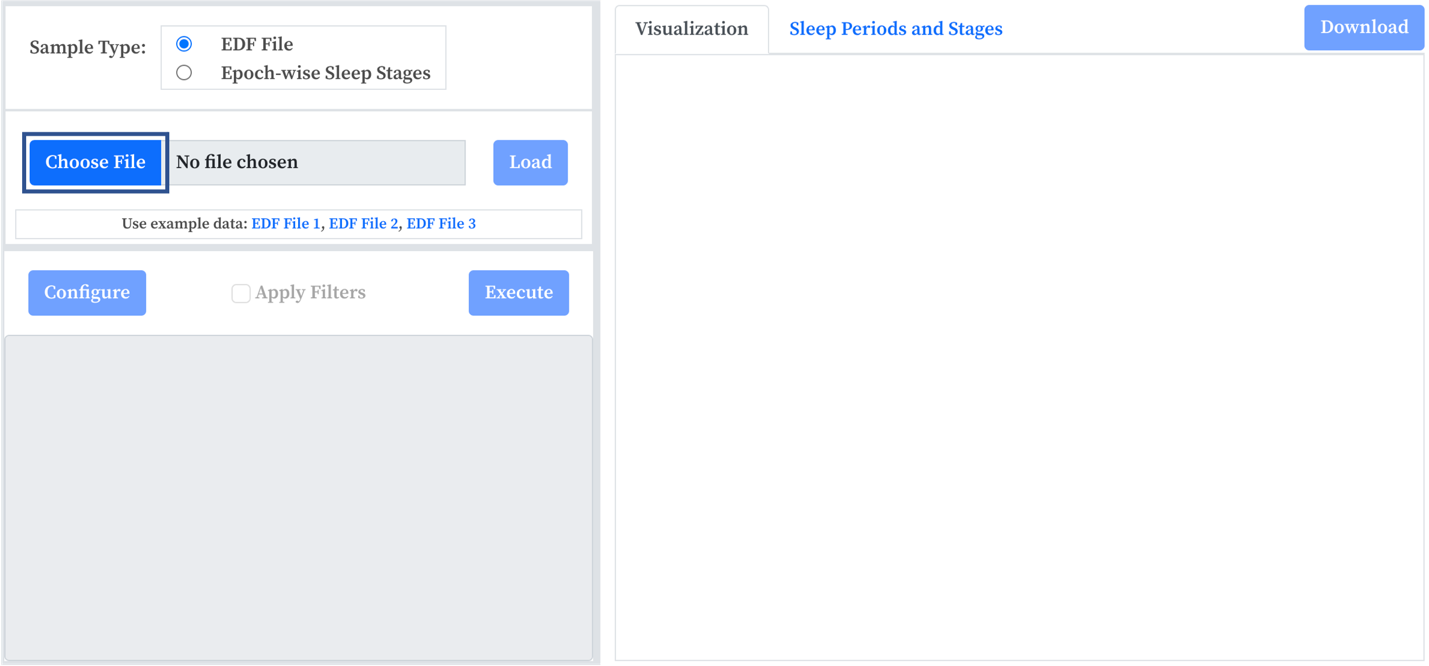**  (Fig, S2A) |
| --- |
| **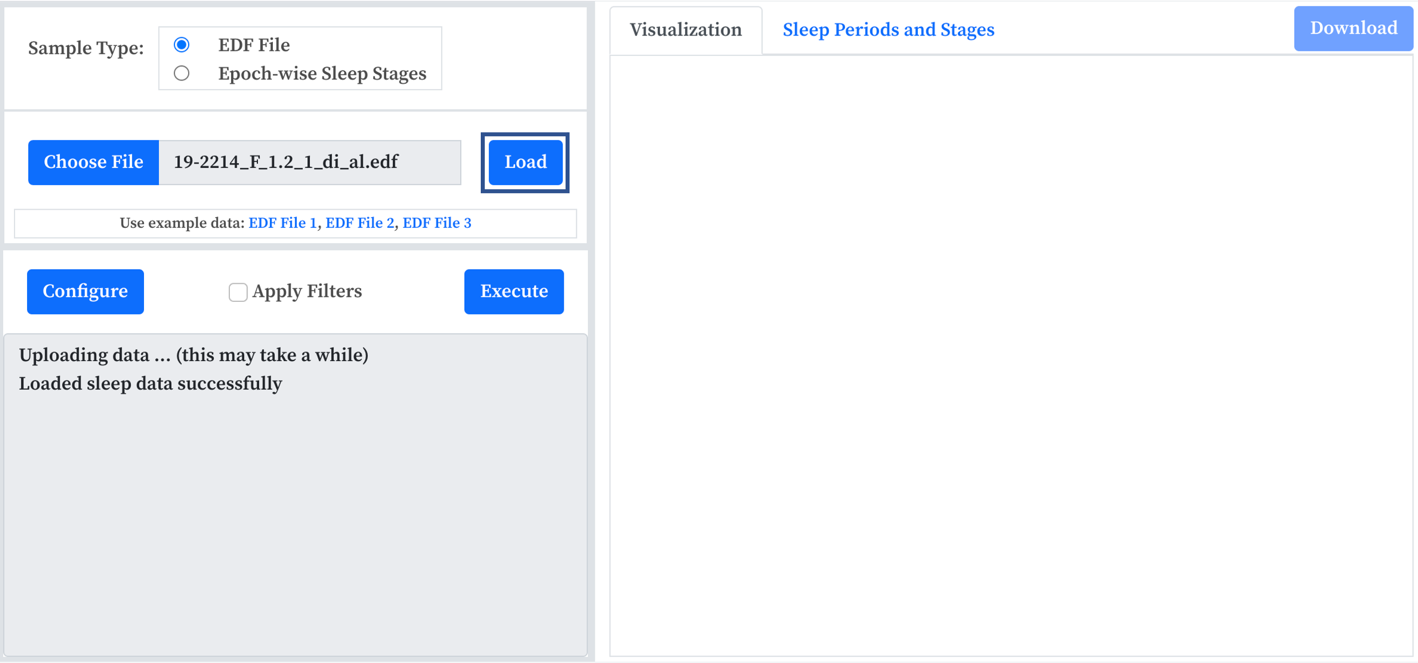**  (Fig. S2B) |
| **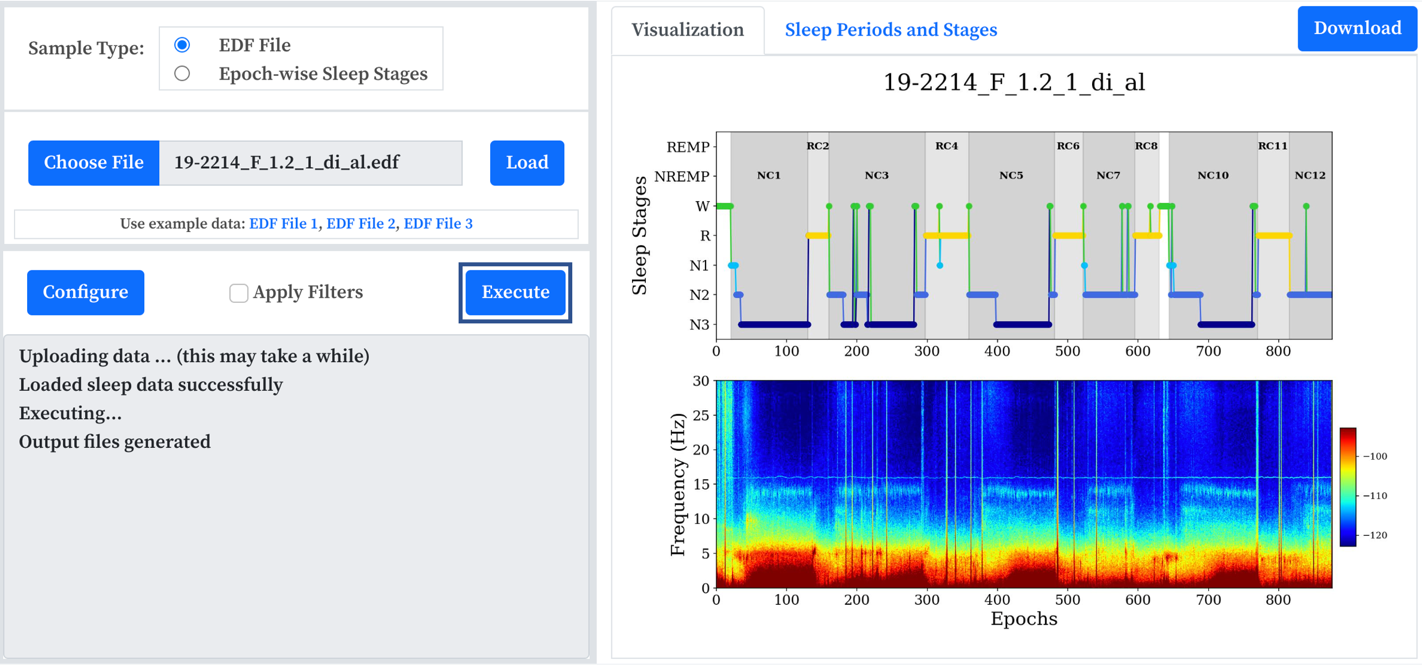**  (Fig. S2C) |
| **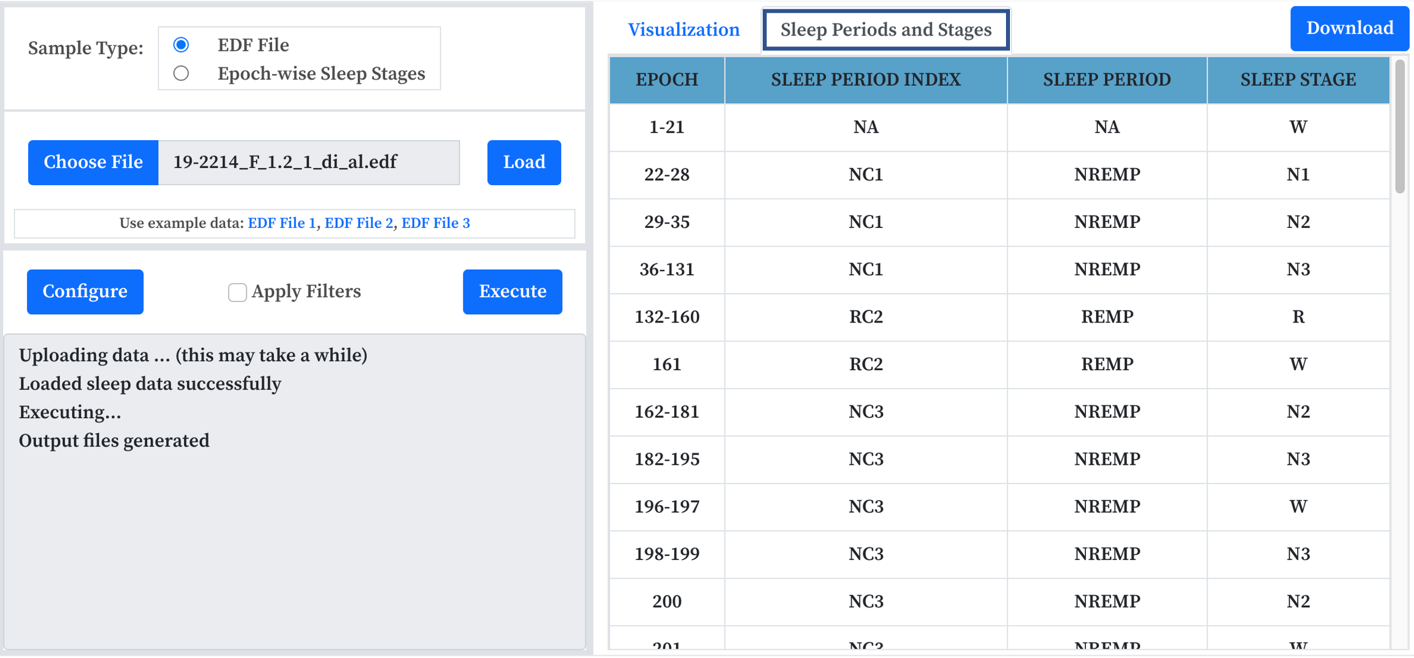**  (Fig. S2D) |
| **Figure S2:** The user interface for the web version of *SSAVE*. **(A)** A user can input the sample data in either the EDF file format or the epoch-based sleep stage annotation file in a text format to the “Sample Path” parameter of the input panel (top half). **(B)** After setting the input parameters, the user can load the data using the “Load” button. After the data are loaded, three other options (“Configure”, “Apply Filter” and “Execute”) will be enabled. The “Configure” button opens the settings window where a user can change various settings (Figure 5) including certain filters. Checking the “Apply Filter” box allows the user to apply the filters to the data before clicking on “Execute” button. **(C)** After execution completes, the output files will be shown in the output panel (bottom half). This panel generally has two tabs; “Visualization” and “Sleep Periods and Stages”. The “Visualization” tab shows the visualization figure with the sleep stages, sleep periods, and the spectrogram. **(D)** The “Sleep Periods and Stages” tab shows the epoch-based sleep stages extracted from the EDF file and the sleep periods identified by *SSAVE*. |

| 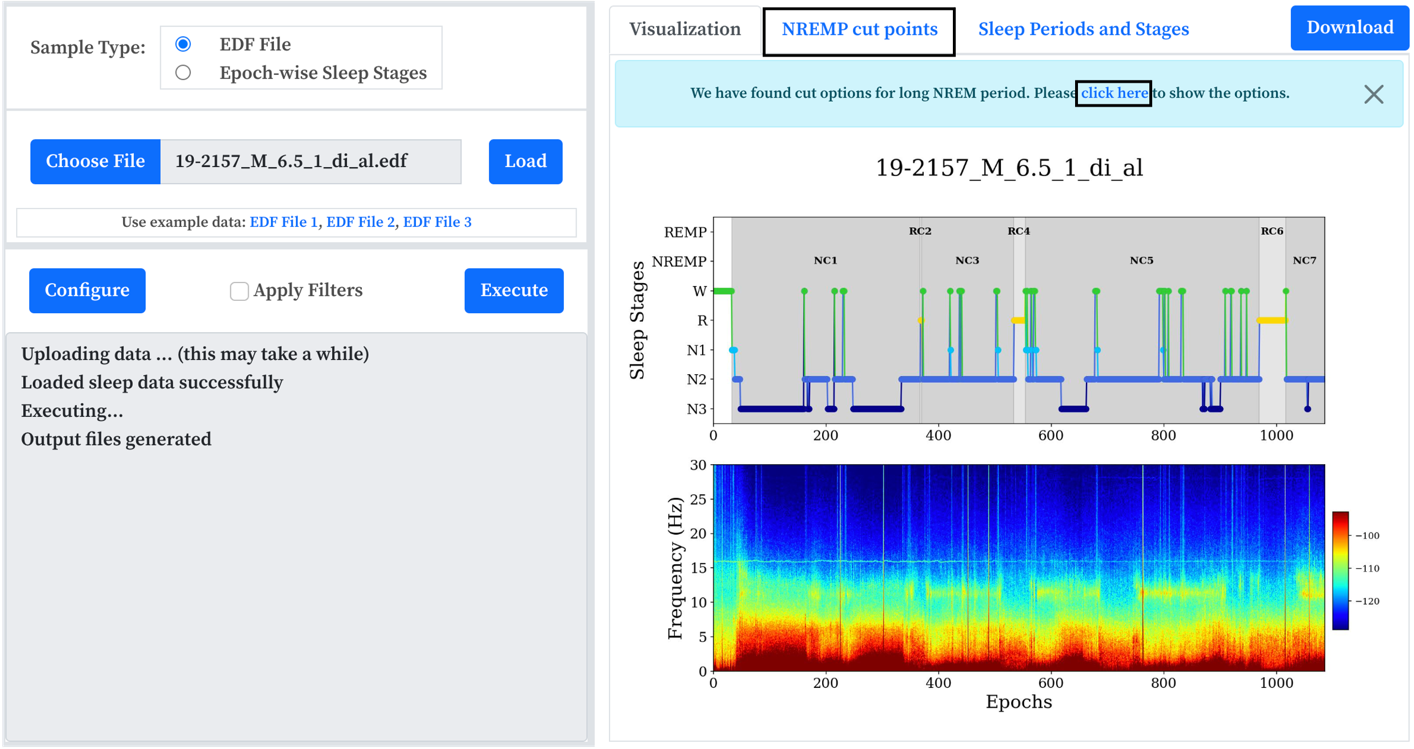  (Fig. S3A) |
| --- |
| **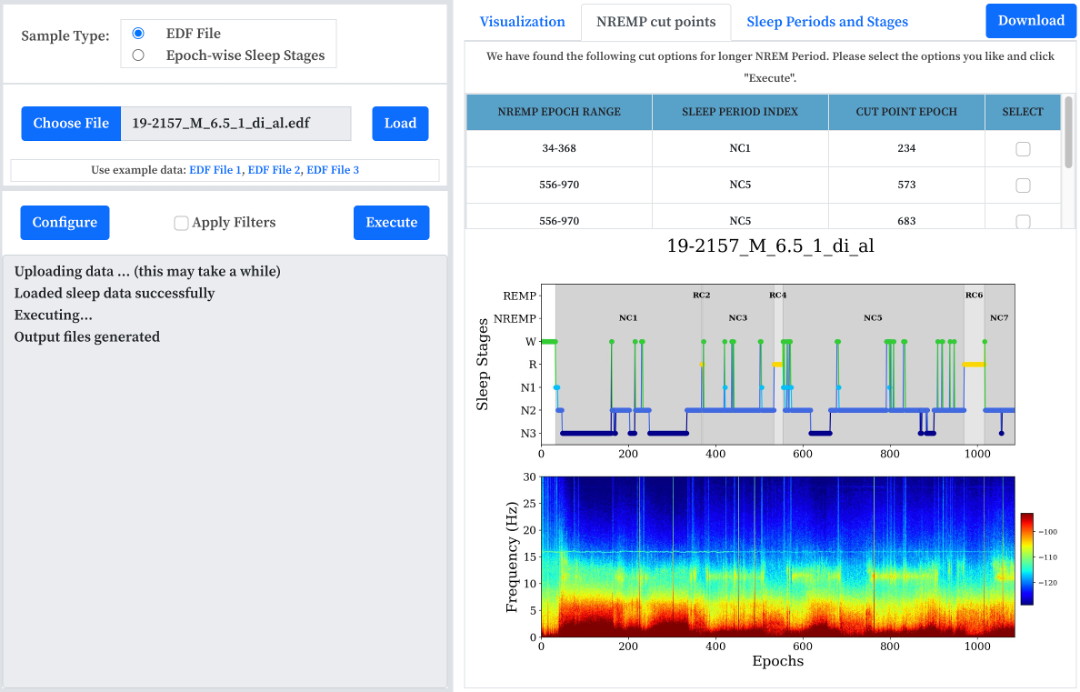**  (Fig. S3B) |
| 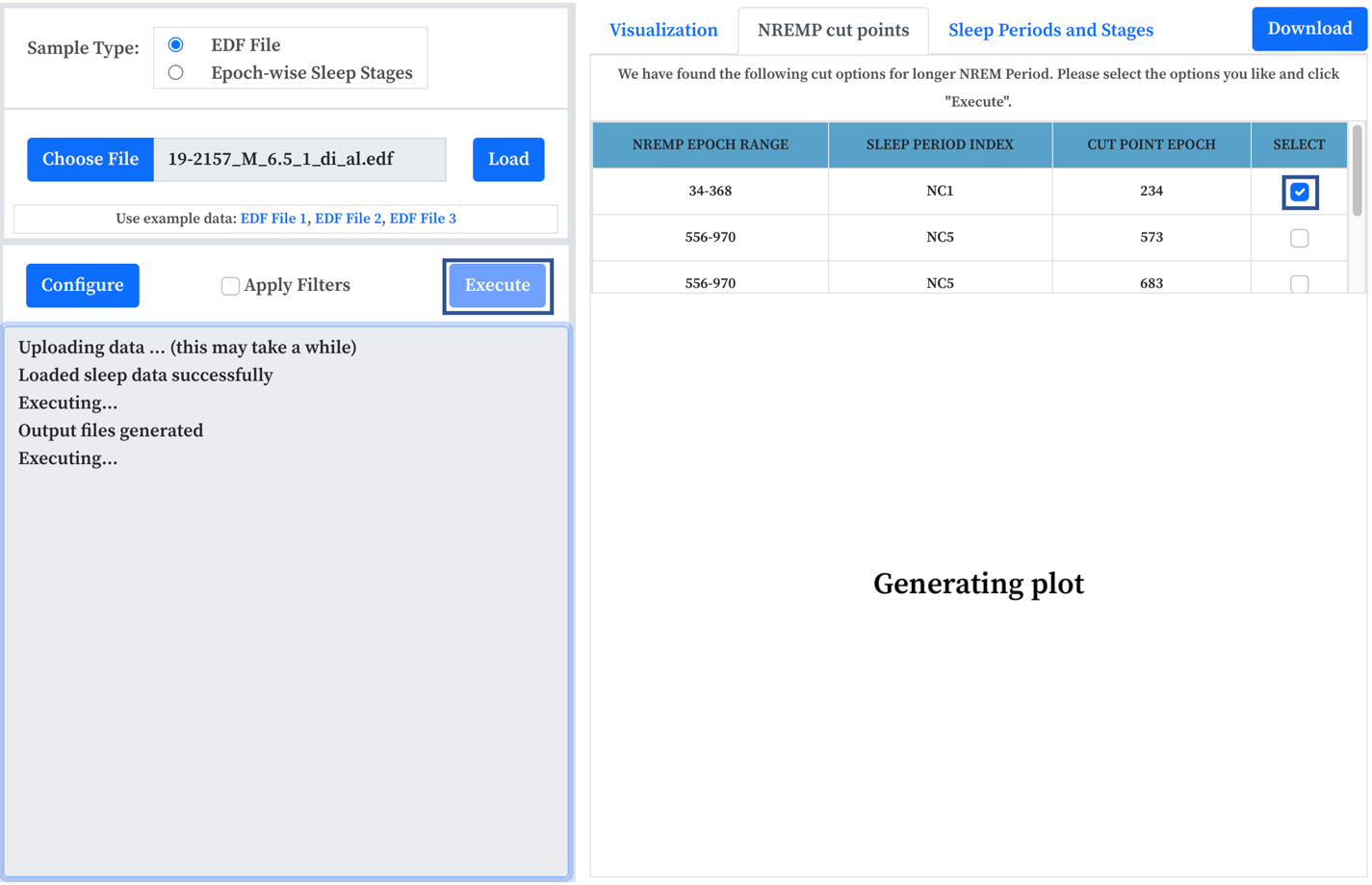  (Fig. S3C) |
| 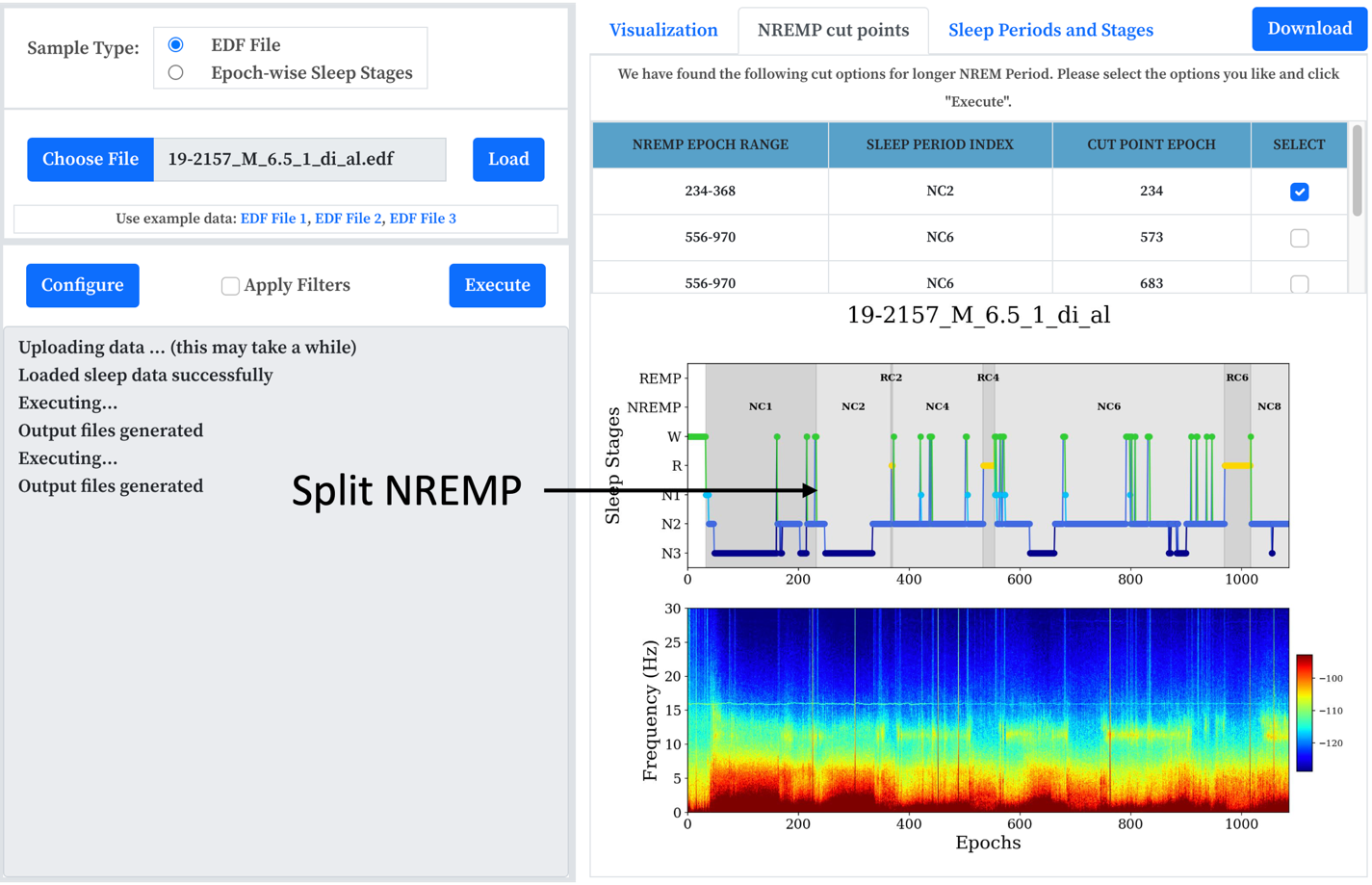  (Fig. S3D) |
| **Figure S3:** The user interface to split a long NREM period. **(A)** When a sleep input file with any long NREM periods is processed, *SSAVE* finds split position options for all of them. If any split option is found, the output panel shows another tab named “NREMP cut points” with all the split options. **(B, C)** In the “NREMP cut points” tab, the user can choose the epochs where the long NREM period will be split and click “Execute” to apply them to the output. **(D)** After clicking the “Execute” button, the selected split options will be applied to the visualization and the output listings. |

| 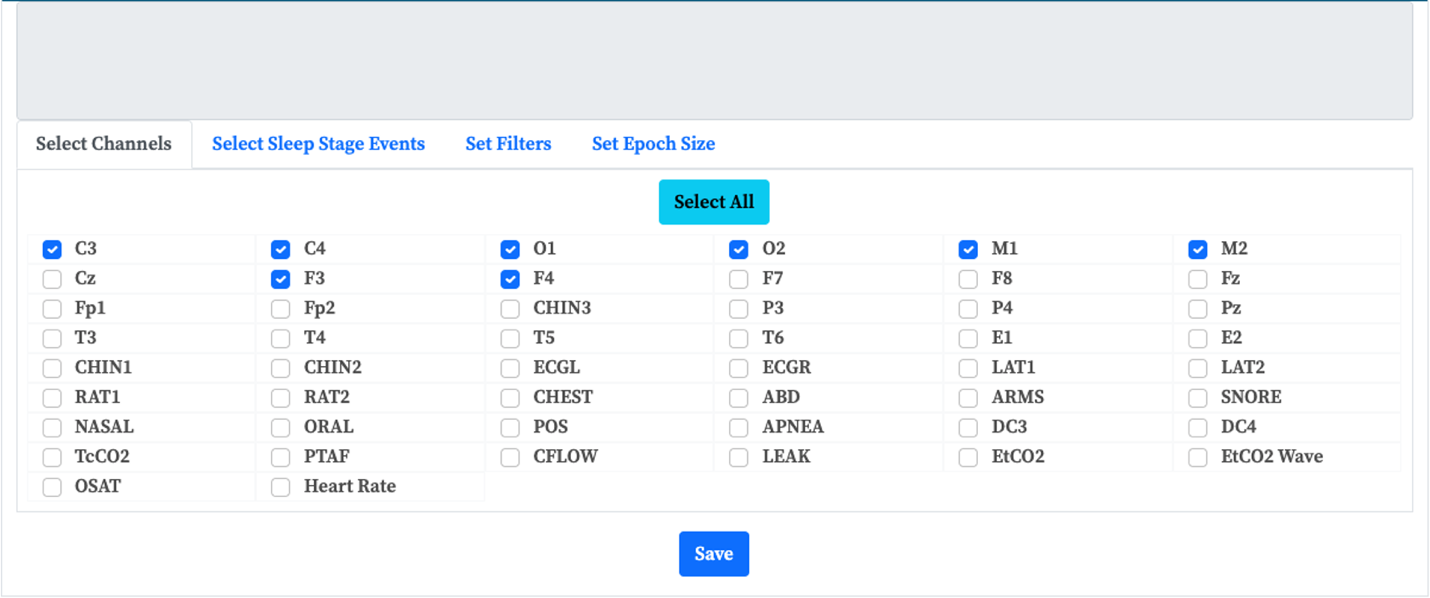  (Fig. S4A) |
| --- |
| 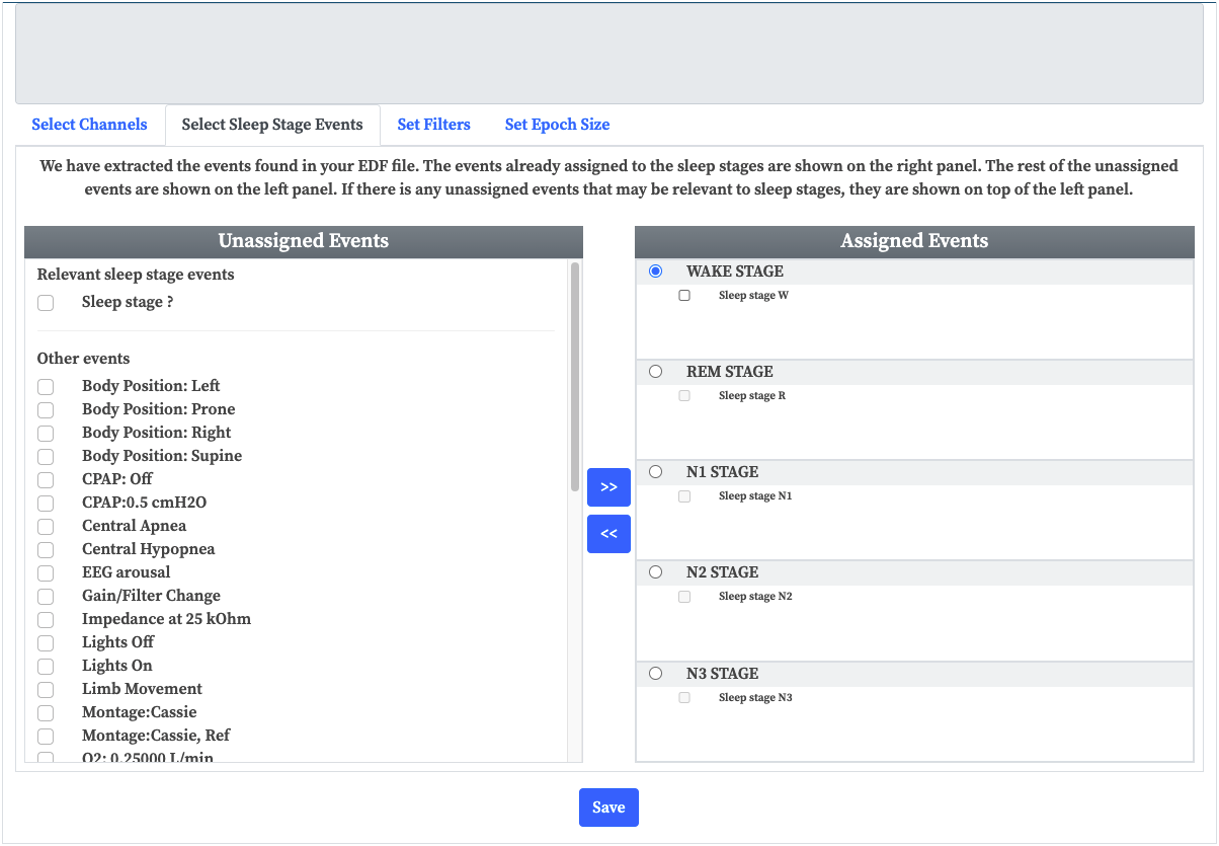  (Fig. S4B) |
| 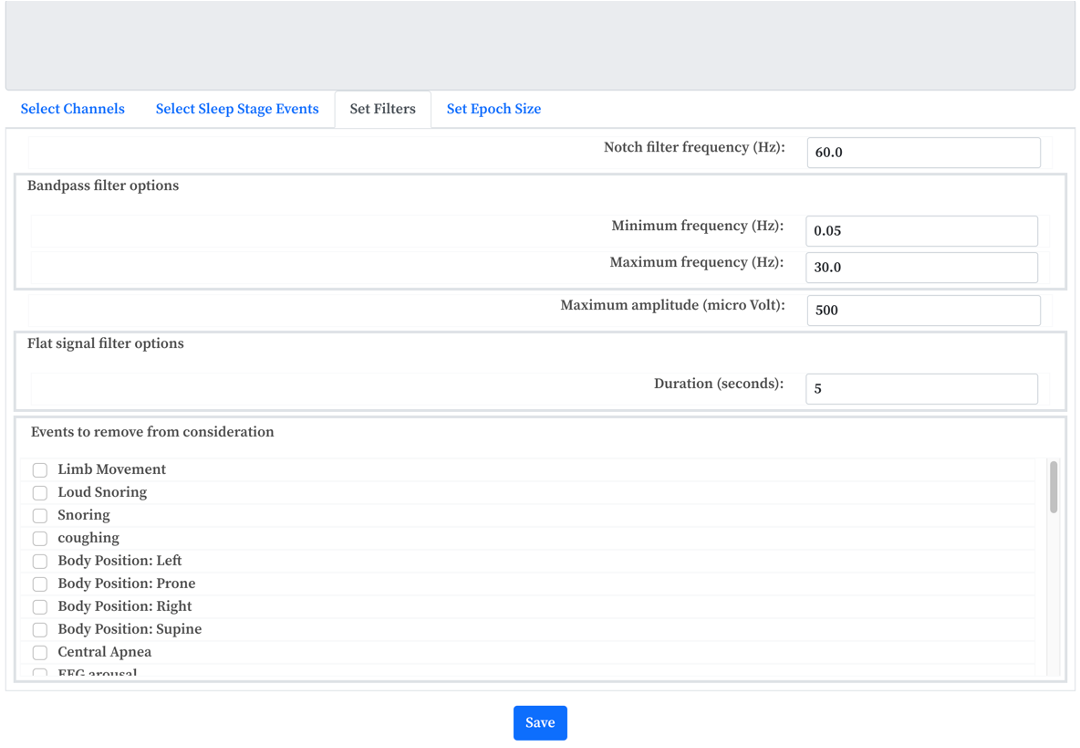  (Fig. S4C) |
| 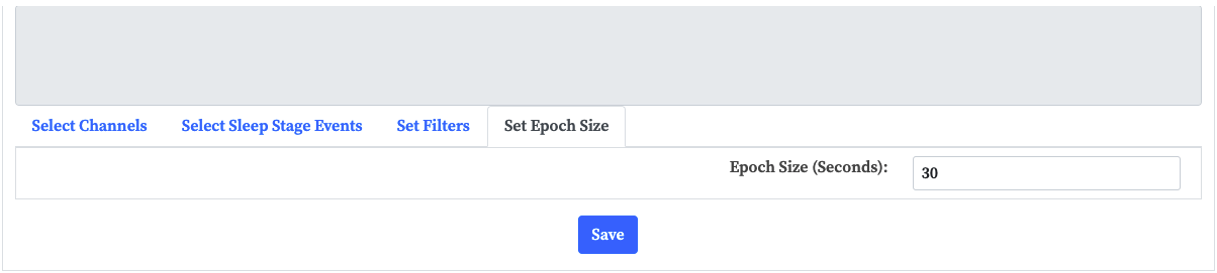  (Fig. S4D) |
| **Figure S4:** The settings interface for the *SSAVE* web application. The settings window has four tabs. **(A)** The “Select Channels” tab shows all the available and selected channels extracted from the user inputted EDF file. The user can select their preferred channels for analysis in this tab. **(B)** The “Select Sleep Stages” tab shows all the available annotations extracted from the EDF file under the left-side “All Annotations” panel of the tab. The default sleep stages extracted from the annotations on the left panel are shown on the right-side panel. The user can add or delete annotations for the sleep stages in this tab. **(C)** The “Set Filters” tab allows the user to apply various filters to the data. **(D)** User can change the size of an epoch in the “Set Epoch Size” tab. Any changes made in these four tabs can be saved by clicking the “Save” button. |

| 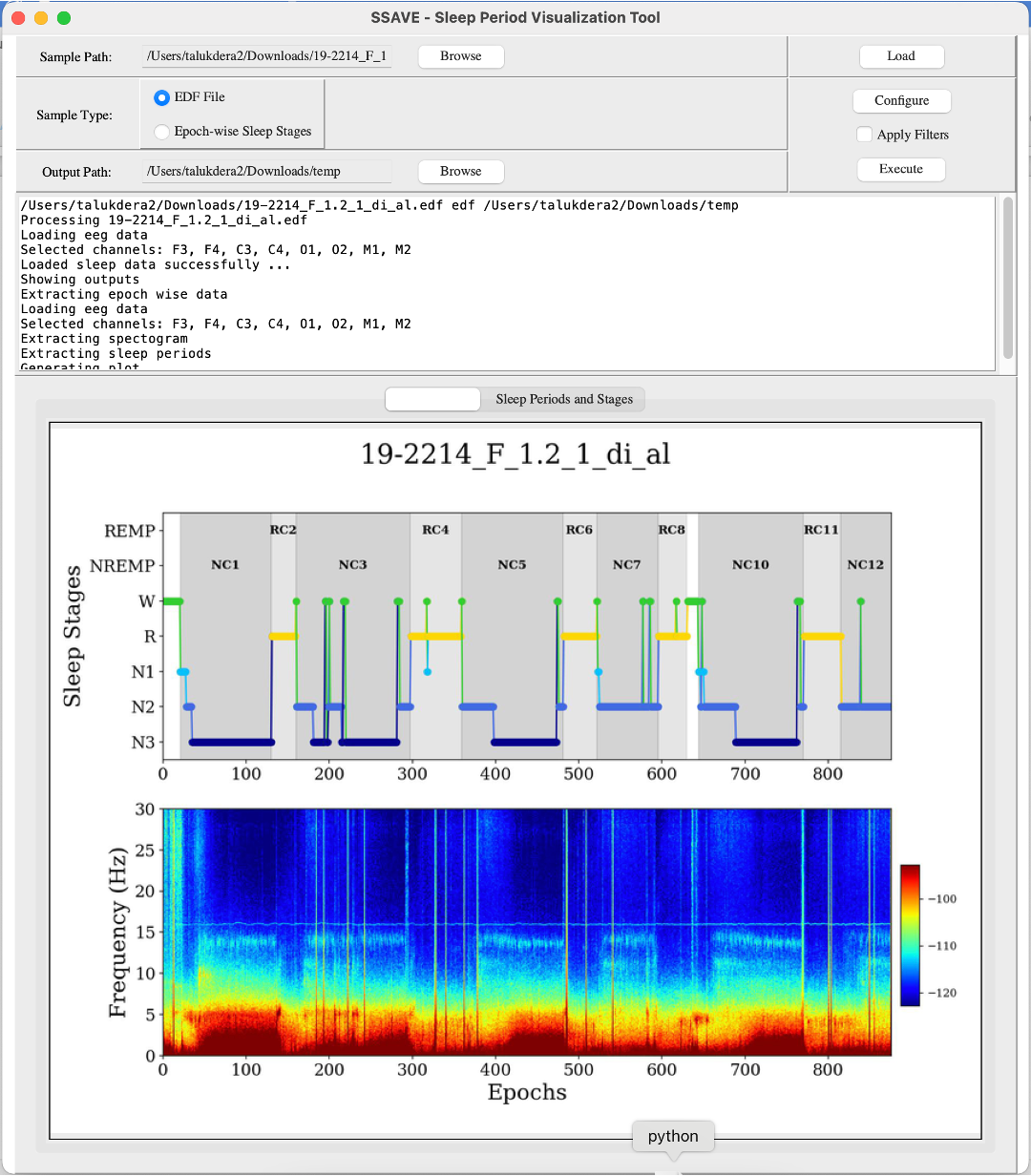  (Fig. S5A) |
| --- |
| 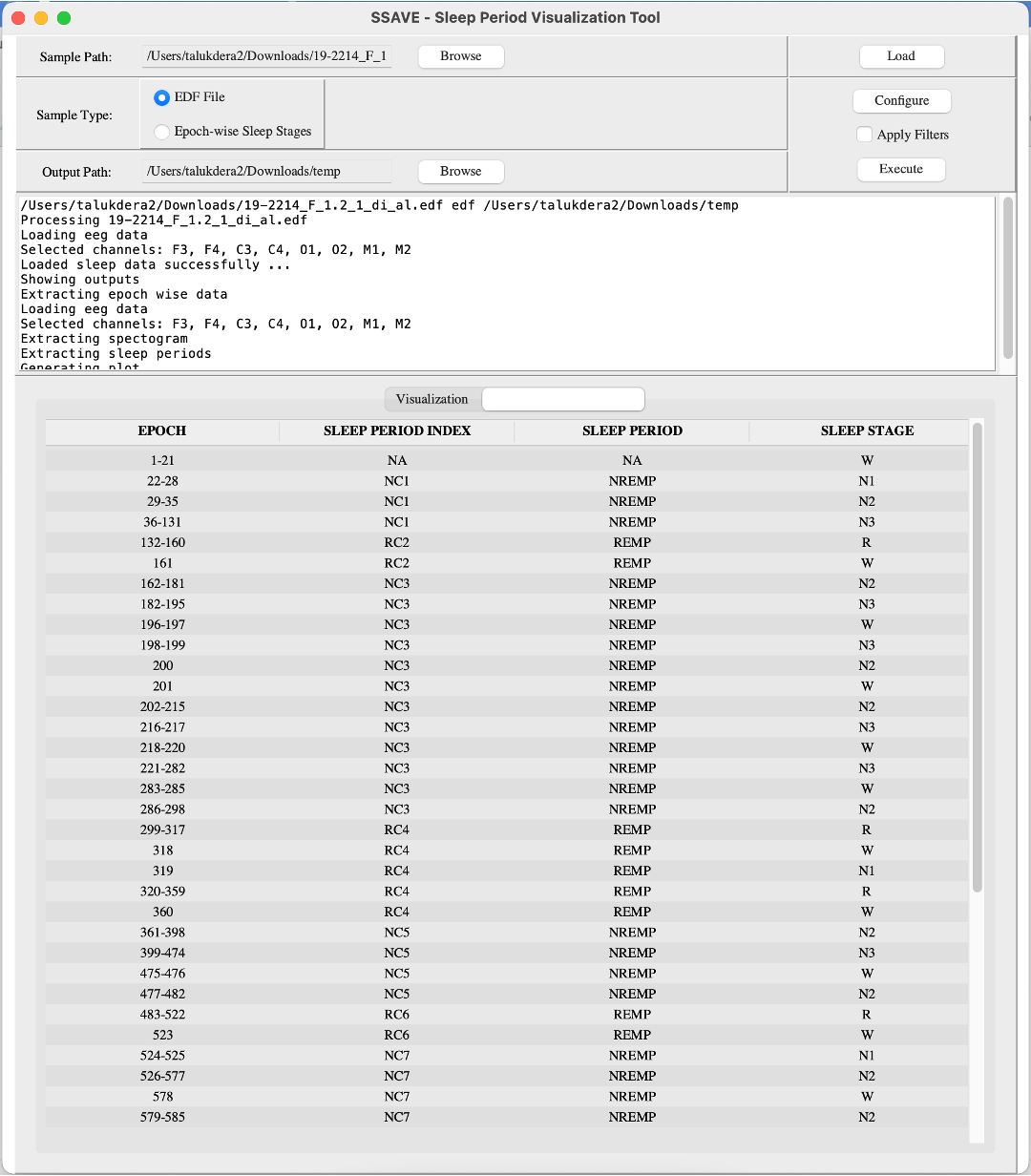  (Fig. S5B) |
| **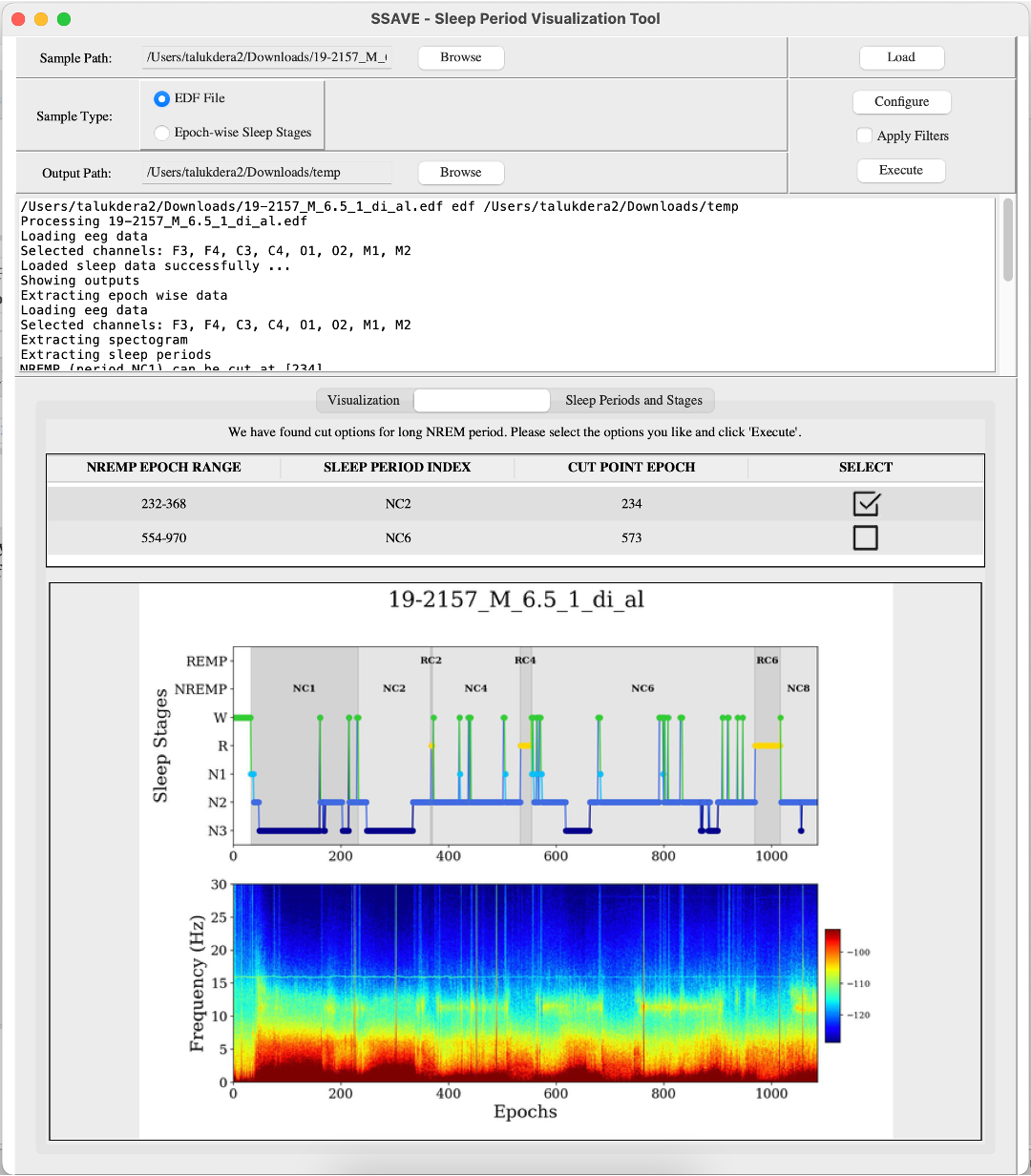**  (Fig. S5C) |
| **Figure S5:** The user interface for the desktop version of *SSAVE*. The output panel generally has two tabs; “Visualization” and “Sleep Periods and Stages”. Another tab named “NREMP Cut Options” appears when an NREMP is longer than maximum duration and needs to be split. **(A)** The “Visualization” tab shows the hypnogram with the sleep stages, sleep periods, and the spectrogram. **(B)** The “Sleep Periods and Stages” tab shows the epoch-based sleep stages extracted from the EDF file and the sleep periods identified by *SSAVE*. **(C)** In the “NREMP Cut Options” tab, the user can choose the epochs where the long NREM period will be split and click “Execute” to apply them to the outputs. After clicking the “Execute” button, the selected split options will be applied to the visualization and the output listings. |

| 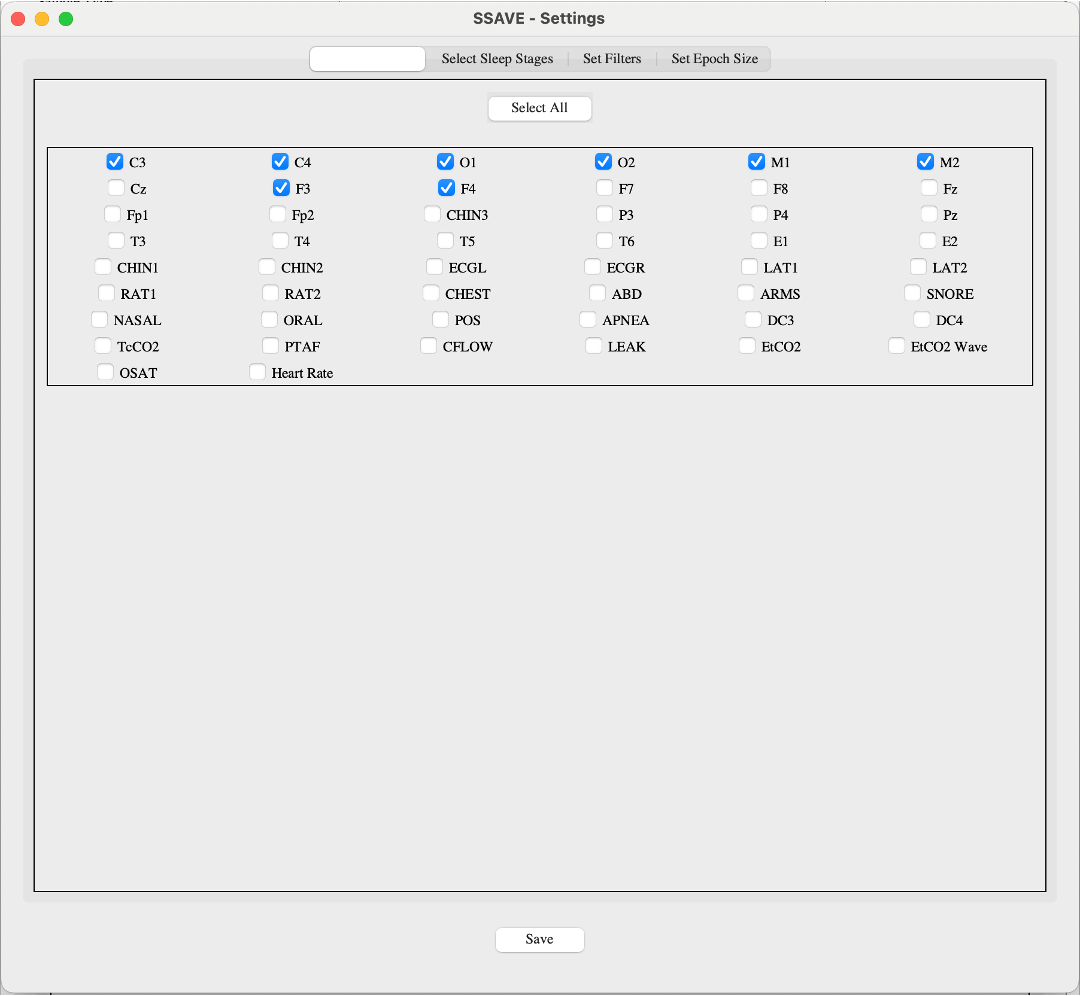  (Fig. S6A) |
| --- |
| 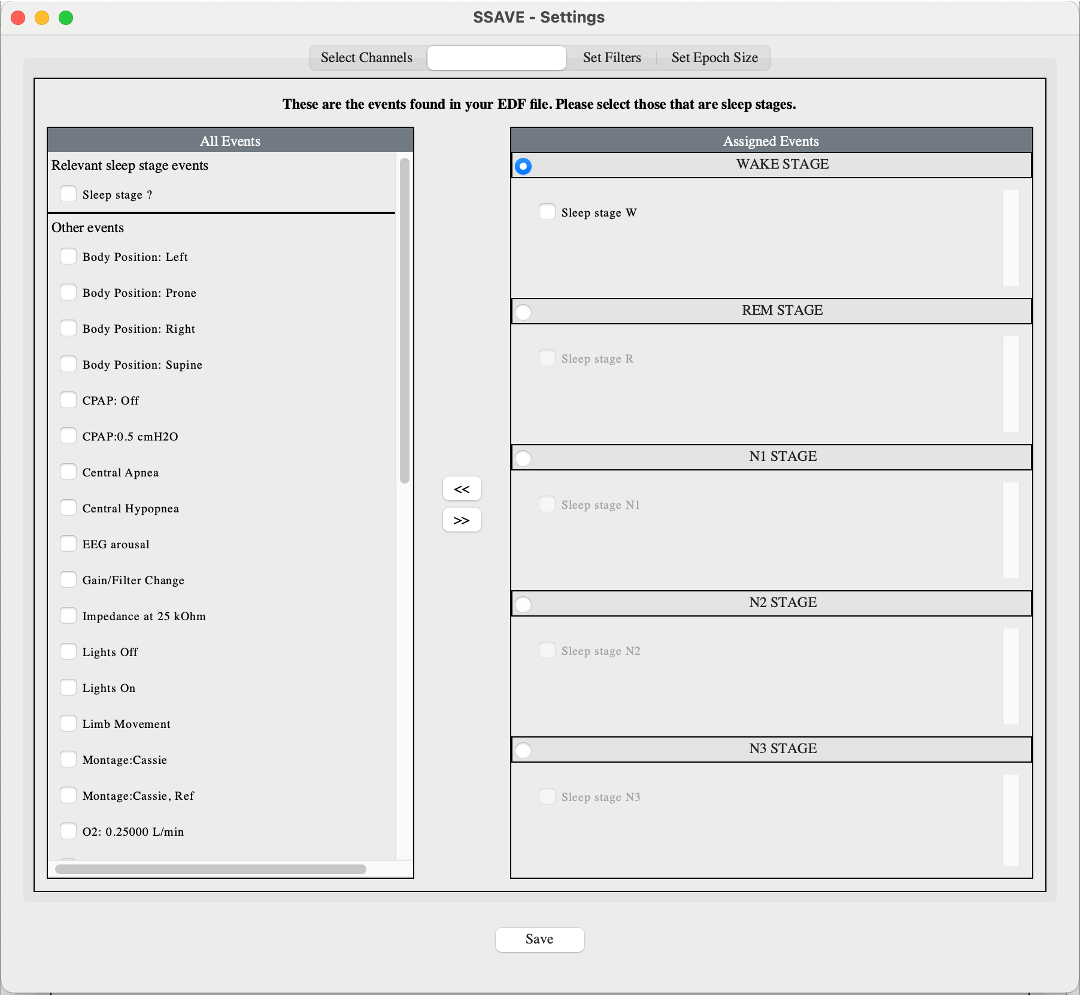  (Fig. S6B) |
| **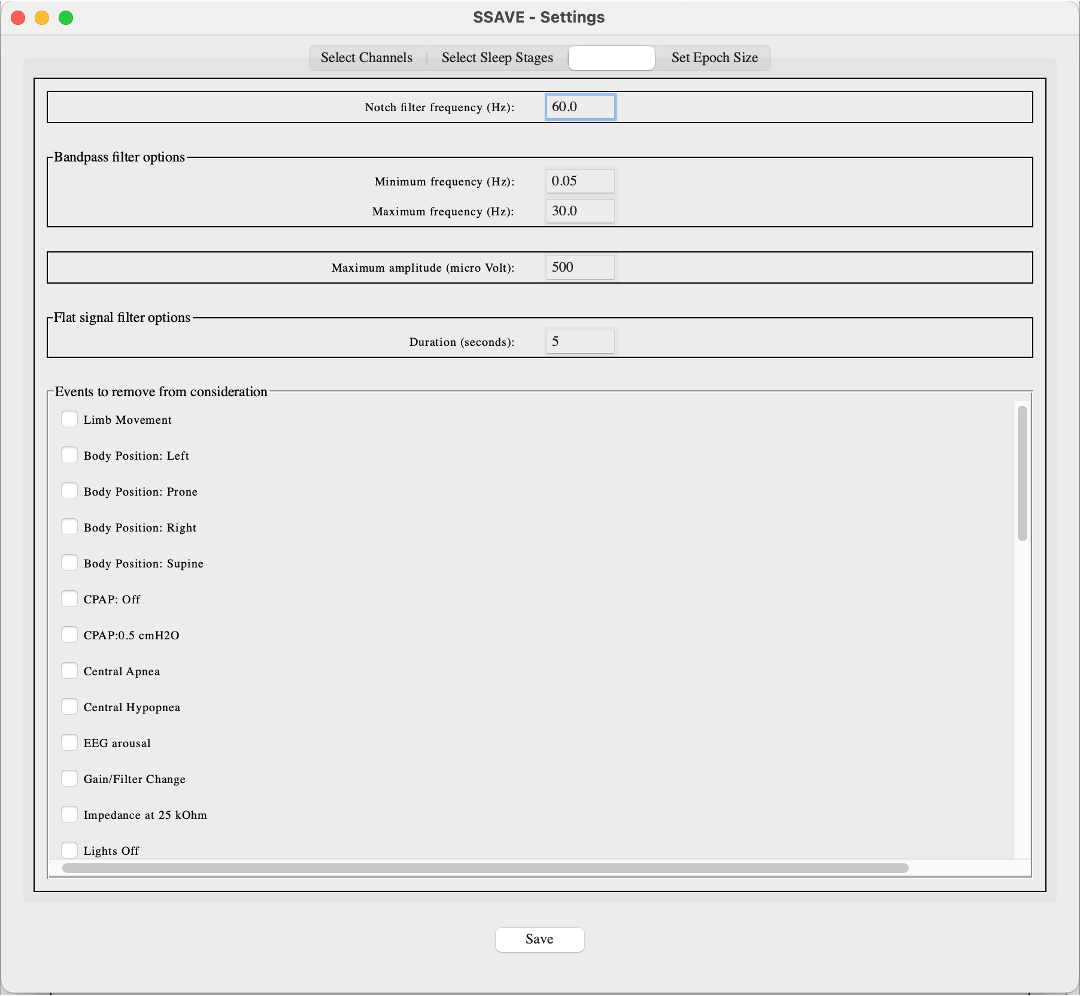**  (Fig. S6C) |
| **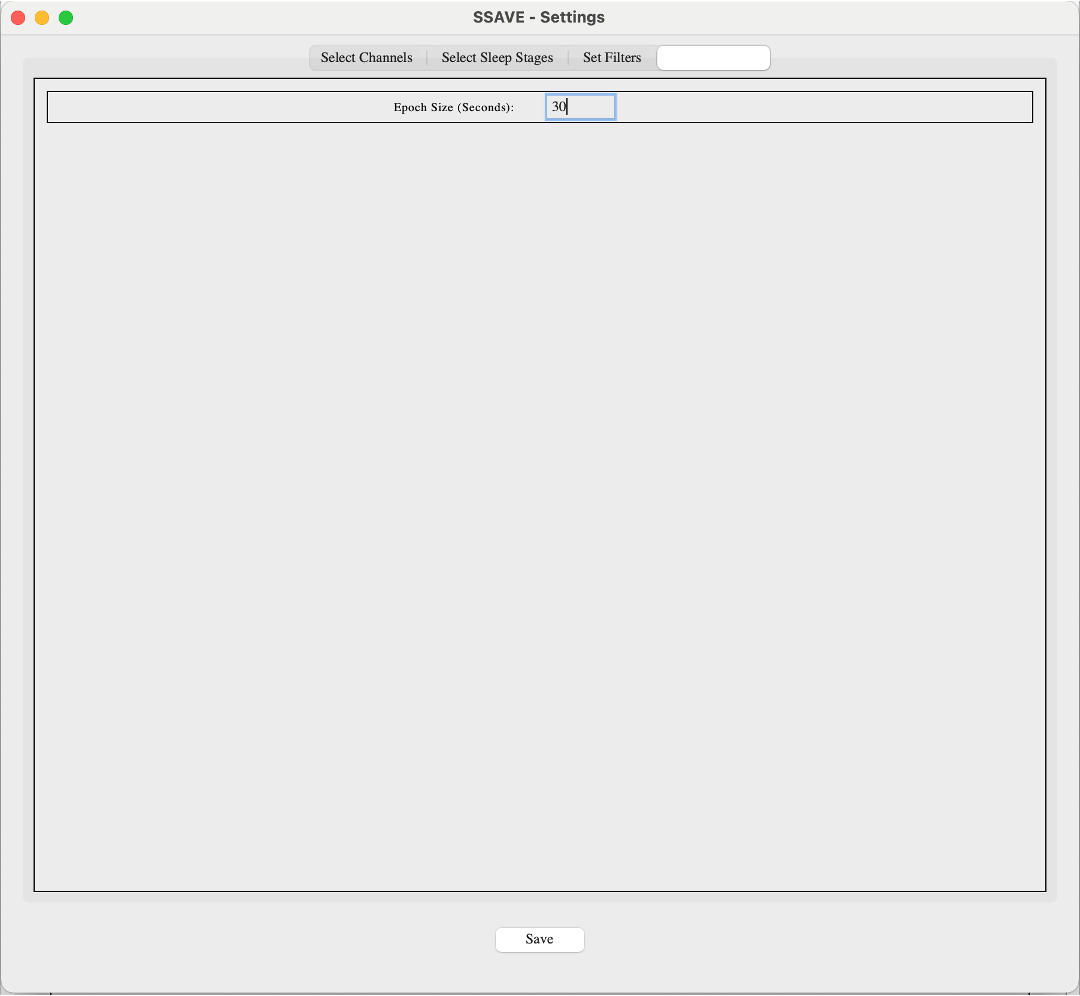**  (Fig. S6D) |
| **Figure S6:** The settings interface for the *SSAVE* desktop application. The settings window has four tabs. **(A)** The “Select Channels” tab shows all the available and selected channels extracted from the user inputted EDF file. The user can select their preferred channels for analysis in this tab. **(B)** The “Select Sleep Stages” tab shows all the available annotations extracted from the EDF file under the left-side “All Annotations” panel of the tab. The default sleep stages extracted from the annotations on the left panel are shown on the right-side panel. The user can add or delete annotations for the sleep stages in this tab. **(C)** The “Set Filters” tab allows the user to apply various filters to the data. **(D)** User can change the size of an epoch in the “Set Epoch Size” tab. Any changes made in these four tabs can be saved by clicking the “Save” button. |

**References**

Prerau, M. J., et al. (2017). "Sleep neurophysiological dynamics through the lens of multitaper spectral analysis." Physiology **32**(1): 60-92.
